# Protective Prognostic Biomarkers Negatively Correlated with Macrophage M2 Infiltration in Low-Grade Glioma

**DOI:** 10.1155/2022/3623591

**Published:** 2022-04-08

**Authors:** Yunyang Zhu, Zhaoming Song, Zhong Wang, Gang Chen

**Affiliations:** ^1^Department of Neurosurgery, The First Affiliated Hospital of Soochow University, Suzhou 215006, China; ^2^Department of Neurosurgery & Brain and Nerve Research Laboratory, The First Affiliated Hospital of Soochow University, Suzhou 215006, China

## Abstract

Tumor-associated Macrophages (TAMs) play a vital role in the progression of glioma. Macrophage M2 has been confirmed to promote immunosuppression and proliferation of low-grade glioma (LGG). Here, we searched for genes negatively correlated with Macrophages M2 by bioinformatical methods and investigated their protective ability for prognosis. LGG and adjacent normal samples were screened out in TCGA and three GEO datasets. 326 overlapped differentially expressed genes were calculated, and their biological functions were investigated by Go and KEGG analyses. Macrophage M2 accounted for the highest proportion among all 22 immune cells by CIBERSORT deconvolution algorithm. The proportion of Macrophage M2 in LGG was also higher than that in normal tissue according to several deconvolution algorithms. 43 genes in the blue module negatively correlated with Macrophage M2 infiltration were identified by weighted gene coexpression network analysis (WGCNA). Through immune infiltration and correlation analysis, FGFBP3, VAX2, and SHD were selected and they were enriched in G protein-coupled receptors' signaling regulation and cytokine receptor interaction. They could prolong the overall and disease-free survival time. Univariate and multivariate Cox regression analyses were applied to evaluate prognosis prediction ability. Interestingly, FGFBP3 and AHD were independent prognostic predictors. A nomogram was drawn, and its 1-year, 3-year, and 5-year survival prognostic value was verified by ROC curves and calibration plots. In conclusion, FGFBP3, VAX2, and SHD were protective prognostic biomarkers against Macrophage M2 infiltration in low-grade glioma. The FGFBP3 and SHD were independent factors to effectively predict long-term survival probability.

## 1. Introduction

With decades of worldwide study, the management of low-grade glioma (LGG) remains controversial. LGG is a progressive and invasive disease of the central nervous system [1]. Although LGG grows relatively slowly, the diffuse and infiltrative characteristics means it could hardly be cured. Malignant transformation to high-grade glioma would inevitably occur, and the mean survival is less than 10 years [2, 3]. Molecular parameters had been incorporated into glioma classification and diagnosis by neuropathology guidelines established in 2014 [4]. Subsequently, 2016 and 2021 World Health Organization (WHO) brain tumor classifications increasingly highlighted the role of molecular diagnostics, although histology and immunohistochemistry remained basic [5, 6]. Apart from surgical operation, the Temozolomide, adjuvant PCV chemotherapy or radiotherapy, immunotherapies including vaccine therapies, immune checkpoint blockade, and chimeric antigen receptor (CAR) T cells were promising therapies for glioma [7, 8]. In view of evidence-based and personalized medicine, individualized biomarkers play important roles in the specific treatment, so we aimed to find protective prognostic biomarkers of LGG to assistant clinical diagnostics and therapy.

Tumor-associated Macrophages (TAMs), which are essential in the tumorigenesis, angiogenesis, and metastasis of glioma [9, 10], account for 30-50% of all noncancerous cells in glioma-immune microenvironment [11]. Microglia/Macrophages, the main component of glioma TAM, have been categorized as M1 polarized (“classically activated”), M2 polarized (“alternatively activated”), and nonpolarized M0 Macrophages [12, 13]. Macrophages M1 promote Th1 immune response and produce proinflammatory cytokines to exert antitumor effects [9, 14]. However, in tumor immune microenvironment, Macrophages M1 transform into M2 activated by interleukin-4, -10, -13 (IL-4, IL-10, and IL-13), colony-stimulating factor-1 (CSF-1), and tumor growth factor *β* (TGF-*β*) [11, 15]. Macrophages M2 promote glioma progression, angiogenesis, and immunosuppression by JAK2/STAT3 signaling, IL-10, IL-6, and IGFBP1 [11, 16]. Expressions of Macrophages M1 were negatively correlated with glioma WHO grade, while M2 showed a positive correlation with pathological grade along with poorer survival [17].

Through seeking genes negatively associated with M2 infiltration in LGG, we expected to find biomarkers for prognosis evaluation and offer potential targeted genes for immune microenvironment therapy.

## 2. Materials and Methods

### 2.1. Data Acquisition and Processing

Datasets with gene expression and clinical information profiles numbered GSE68848, GSE4290, and GSE16011 were downloaded from the Gene Expression Omnibus (GEO) database. After removing high grade glioma and pathological lacking samples, 95 LGGs and 28 adjacent normal samples were left in GSE68848, 45 LGGs and 23 adjacent normal samples were left in GSE4290, and 32 LGGs and 8 adjacent normal samples were left in GSE16011. In addition, 516 primary LGGs and 5 normal samples were chosen from The Cancer Genome Atlas (TCGA) database. Limma package in R [18] was used for data rectification. The clinical information was presented in Supplement File 1.

### 2.2. Differential Expression Analysis

Differential genes (DEGs) were calculated by DESeq2 package in R [19]. Cut off value of ∣log2 Fold Change (FC) | ≥1 and adj. *p* < 0.05 was defined as the differentially expressed genes in GSE68848, GSE4290, and GSE16011. In TCGA dataset, the cut off value was set to ∣log2 (FC) | ≥1 and adj. *p* < 0.05.

### 2.3. Enrichment Analysis

Gene functions were enriched in three parts, respectively, by Gene Ontology (Go) analysis: cellular component (CC), molecular function (MF), and biological process (BP). Kyoto Encyclopedia of Genes and Genomes (KEGG) database was utilized to analyze biological pathways and functions of target genes. Single gene enrichment was achieved by Gene Set Enrichment Analysis (GSEA). R package clusterProfiler [20] was applied for enrichment analysis.

### 2.4. Evaluation of Immunocyte Infiltration

A deconvolution algorithm based on gene expression called CIBERSORT [21] was applied to evaluate the proportion of 22 infiltrating immune cells. The leukocyte signature matrix (LM22) and expression matrix of datasets were input to calculate the immunocyte proportion in tumor and normal samples by CIBERSORT.R. Moreover, the “IOBR” package [22] was used to analyze the Macrophage levels by xCell, EPIC, and ssGSEA methods.

### 2.5. Weighted Gene Coexpression Network Analysis (WGCNA)

The coexpression genes of Macrophage M2 were got by weighted gene coexpression network analysis with R package termed “WGCNA” [23]. Samples were clustered by a hierarchical clustering algorithm implemented in R function “hclust.” The soft thresholding power *β* = 8 was selected by R function “pickSoftThreshold” (scale free *R*^2^ = 0.85). The expression matrix was converted into the adjacent matrix and then into the topological matrix for gene clustering. Average linkage hierarchical cluster approach was utilized to cluster genes in a dendrogram. The dynamic tree shear algorithm was also applied to determine gene module assignment. Module eigengenes (MEs) were calculated by R function “moduleEigengenes.” Gene significance (GS) and module membership (MM) were defined as the correlation value. Pearson correlation coefficients between them were calculated.

### 2.6. Immunocyte Infiltration and Survival Analysis

Cox Proportional Hazard Models of immunocyte in LGG and GBM were got from TIMER (Tumor Immune Estimation Resource) database (https://cistrome.shinyapps.io/timer/). Correlations between gene expression and immunocyte infiltration in LGG were also evaluated by TIMER database. Survival information and expressions of single gene among GBM, LGG and normal tissue were obtained from Gene Expression Profiling Interactive Analysis (GEPIA) database (http://gepia.cancer-pku.cn).

### 2.7. Construction of Prognostic Model

The prognostic model was constructed on TCGA-LGG dataset due to the extensive and complete survival data in it. Primary LGG samples in TCGA database were randomly divided into a training dataset (*n* = 256) and a testing set (*n* = 256). In the training dataset, univariate Cox proportional hazards regression analysis was used to verify the prognosis predictability with the cut-off of *p* value < 0.05. Then, a multivariate Cox proportional hazards regression analysis was applied to further filter these candidate genes and the Akaike information criterion (AIC) was used to avoid overfitting. The risk scores were calculated based on the following formula by “survival” package in R. The median risk score was used as the cut-off value to distinguish high and low risk groups. Kaplan-Meier curves and time-dependent ROC curves were used to analyze its ability of prognosis prediction.

(1) $$\begin{array}{c} \text{Risk score}=h\left(t\right)=h_{0}\left(t\right)\times \exp\left(\sum \beta_{i}X_{i}\right). \end{array}$$

In the risk score formula, “*h*(*t*)” represents the risk function at “*t*” time and “*h*_0_(*t*)” was the baseline risk function when all covariates'values were zero at “*t*” time. “*X*” was the expression of each prognostic gene and “*β*” represented the coefficients in the multivariate Cox regression model.

### 2.8. Establishment and Analysis of Nomogram Prognosis Model

Risk score was seemed as a single variate in univariate and multivariate Cox regression analysis together with clinical variables such as age and gender in training and testing dataset. Next, all independent prognostic factors were incorporated to construct a nomogram to assess the 1-year, 3-year, and 5-year overall survival (OS) of LGG. Besides, the calibration curve of the nomogram was plotted to estimate the nomogram's predictive ability. Time-dependent ROC curves were drawn to evaluate predictive sensitivities and specificities of prognostic factors.

## 3. Results

### 3.1. Differentially Expressed Genes and Functional Enrichment Analysis

**Figure 1:**
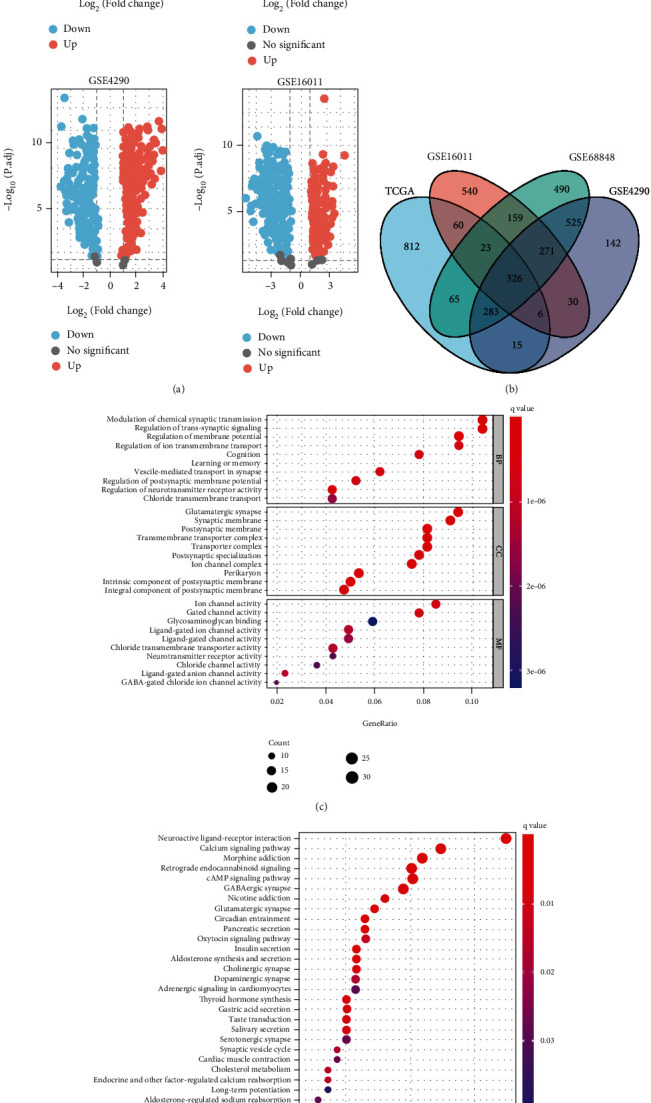
Differential expression analysis and functional enrichment analysis. (a) Volcano plot of differential expression genes of LGG in TCGA, GSE68848, GES4290, and GSE16011 databases. (b) The Venn diagram for overlapping DEGs. (c). GO enrichment analysis (top 10 in each section). BP: biological process; CC: cellular component; MF: molecular function. (d) KEGG enrichment analysis (top 30 KEGG terms).

Differential expression analysis of LGG and adjacent normal tissue in the 4 datasets showed 2142 DEGs in GSE68848, 1598 DEGs in GSE4290, 1415 DEGs in GSE16011, and 1509DEGs in TCGA-LGG (Figure 1(a)). 326 overlapping genes (Figure 1(b)) were chosen for GO and KEGG enrichment analysis (Figure 1(c)). DEGs were enriched in the biological functions such as regulation of transsynaptic signaling, modulation of chemical synaptic transmission, and ion and gated channel activity. DEGs were also involved in the pathways such as neuroactive ligand-receptor interaction and calcium signaling pathway (Figures 1(c) and 1(d)).

### 3.2. Proportion of Macrophage M2

**Figure 2:**
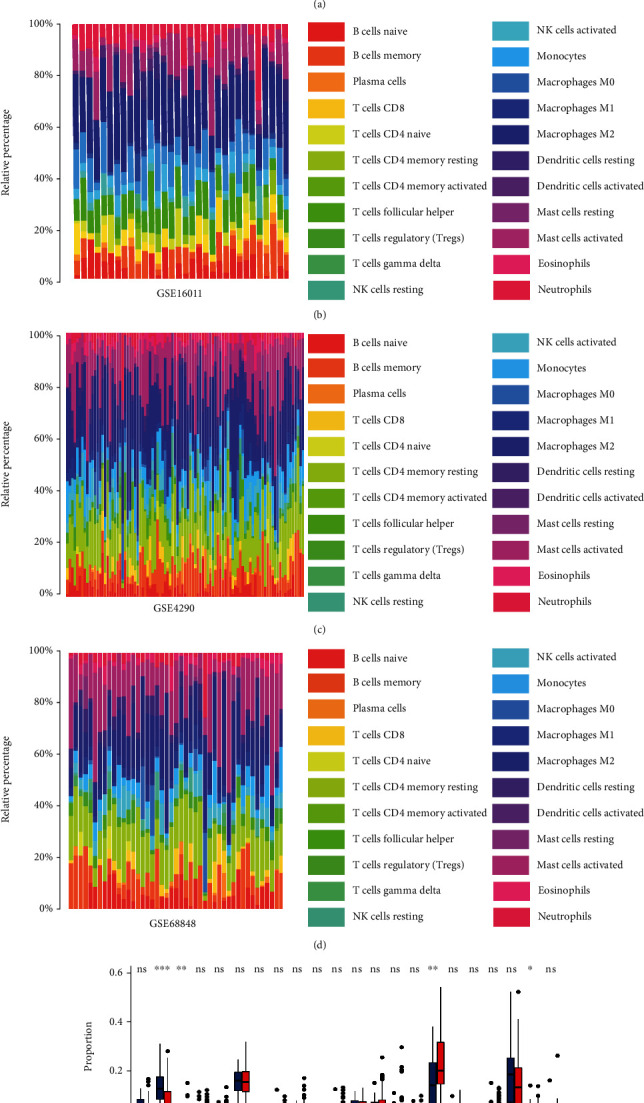
Proportion of 22 immune cells calculated by CIBERSORT. The immunocyte proportion of LGG in the (a) TCGA dataset, (b) GSE16011 dataset, (c) GSE4290 dataset, and (d) GSE68848 dataset. (e) The immunocyte proportion between LGG and adjacent normal tissue in GSE68848 dataset. ^∗^*p* < 0.05, ^∗∗^*p* < 0.01, ^∗∗∗^*p* < 0.001, ns: no statistical significance.

Proportion of 22 immune cells was calculated by CIBERSORT deconvolution algorithm in LGG. Macrophage M2 accounts for the highest proportion among them (Figures 2(a)–2(d). Macrophage M2 counts for 44% ± 12.0% of all immunocytes in TCGA-LGG dataset, 25% ± 6% in GSE16011, 21% ± 10% in GSE4290, and 25% ± 12% in GSE68848 with *p* value < 0.05. The GSE68848 dataset was selected for further WGCNA calculation in which Macrophage M2 proportion was significantly higher than that in adjacent normal tissue (Figure 2(e), *p* < 0.01) by Mann–Whitney *U* test. Moreover, in order to further confirm the result, we used more deconvolution algorithms to estimate Macrophage M2 in GSE68848 dataset, such as xCell, EPIC, and ssGSEA algorithms. Not surprisingly, Macrophage proportion also was higher than that in normal tissue (Figure S1).

### 3.3. Identification of Genes Negatively Correlated with Macrophage M2 by WGCNA

**Figure 3:**
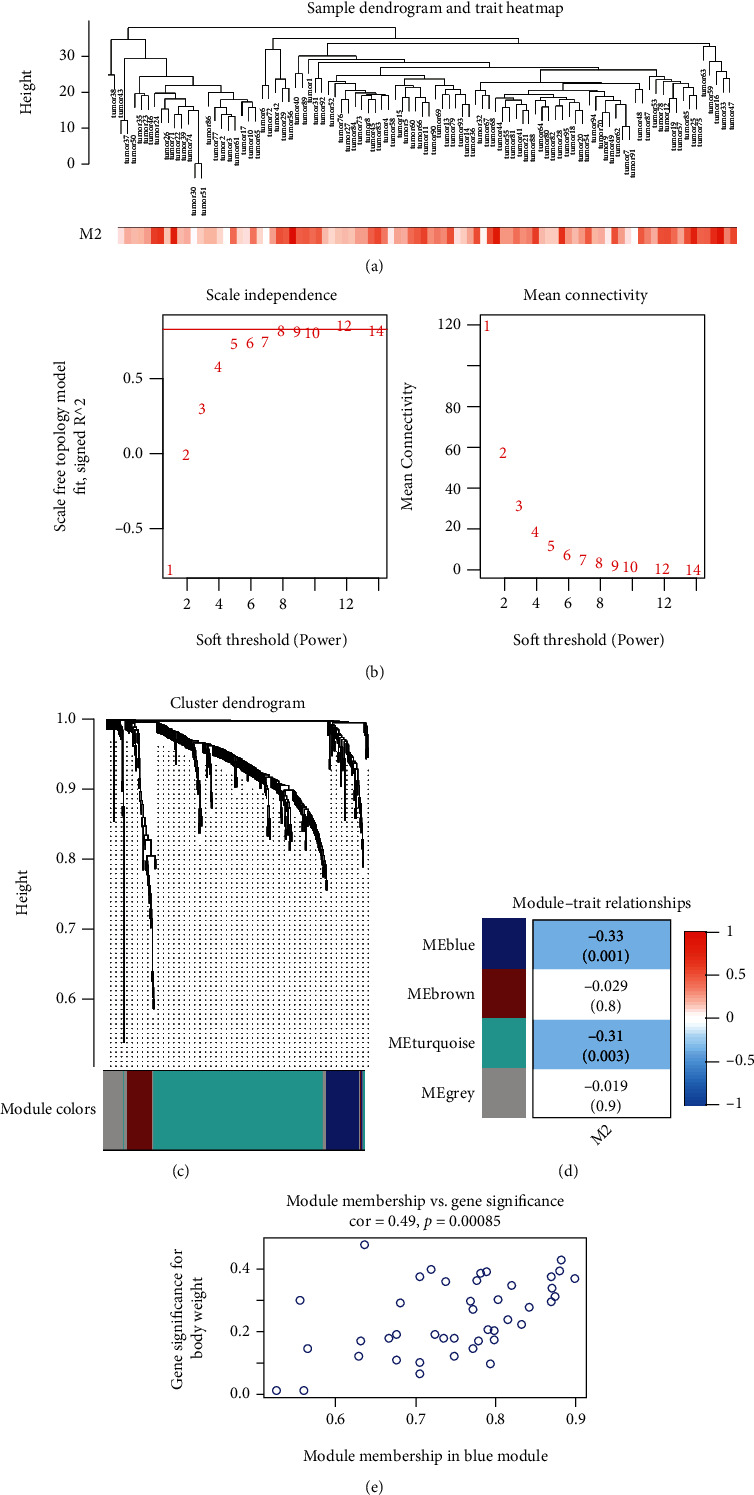
WGCNA analysis. (a) Clustering dendrogram of LGG samples in GSE68848 and heat map of Macrophage M2. (b) Selection of soft thresholding power (optimal *β* = 8). (c) Dendrogram of different modules. (d) The relationship between coexpression modules and Macrophage M2 proportion (the correlation value was labelled in each square, and the corresponding *p* value was labelled in the bracket) (e) The scatter plot of blue module membership vs gene significance (correlation value = 0.49, *p* < 0.001).

The coexpression network (Figure 3(a)) was constructed by 326 DEG expression in 95 LGG samples in GSE68848, along with Macrophage M2 heat map. The optimal soft thresholding power *β* was 8 (Figure 3(b)). Dendrogram of coexpression modules was shown in Figure 3(c), and the genes were classified into 4 different modules. Blue module had the most significant negative correlation with Macrophage M2 in Figure 3(d) (correlation value = −0.33, *p* = 0.001). 43 gene memberships in the blue module were significantly related to gene significance (GS) of M2 Macrophages with a correlation value of 0.49 and *p* value < 0.001 (Figure 3(e)).

### 3.4. Identification of Hub Genes

**Figure 4:**
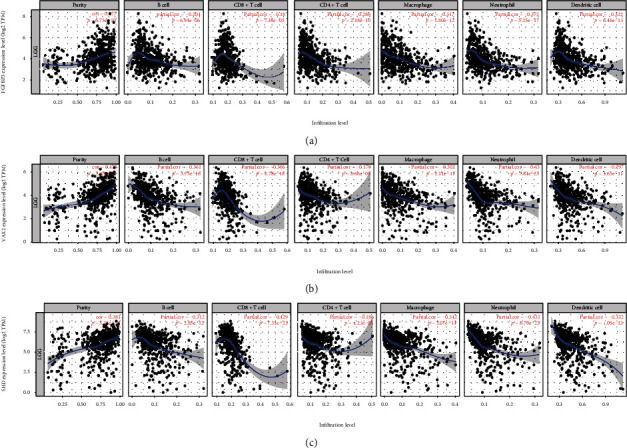
Correlation of tumor purity and immune cell infiltration (CD8+ T cell, CD4+ T cell, Macrophage, B cell, neutrophil, and dendritic cell) with (a) FGFBP3, (b) VAX2, and (c) SHD.(*p* values < 0.001)).

To further select the hub genes which negatively correlated with Macrophage M2, we analyzed the immune infiltration levels in LGG and the correlation of tumor purity with TIMER database. The top five negative correlation genes were FGFBP3, ID4, VAX2, SHD, and STON1. Since genes highly expressed in the immune microenvironment are expected to have negative associations with tumor purity, ID4, and STON1 which had an unsignificant association with tumor purity (*p* > 0.05) were excluded. The hub genes were FGFBP3, VAX2, and SHD. Correlation of tumor purity and immune cell infiltration (B cell, CD8+ T cell, CD4+ T cell, Macrophage, neutrophil, and dendritic cell) is shown in Figure 4. FGFBP3 (correlation value = −0.317, *p* < 0.001), VAX2 (correlation value = −0.302, *p* < 0.001), and SHD (correlation value = −0.342, *p* < 0.001) were all negatively correlated with Macrophage infiltration in LGG.

### 3.5. Survival Analysis of Genes Negatively Correlated with Macrophage M2

**Figure 5:**
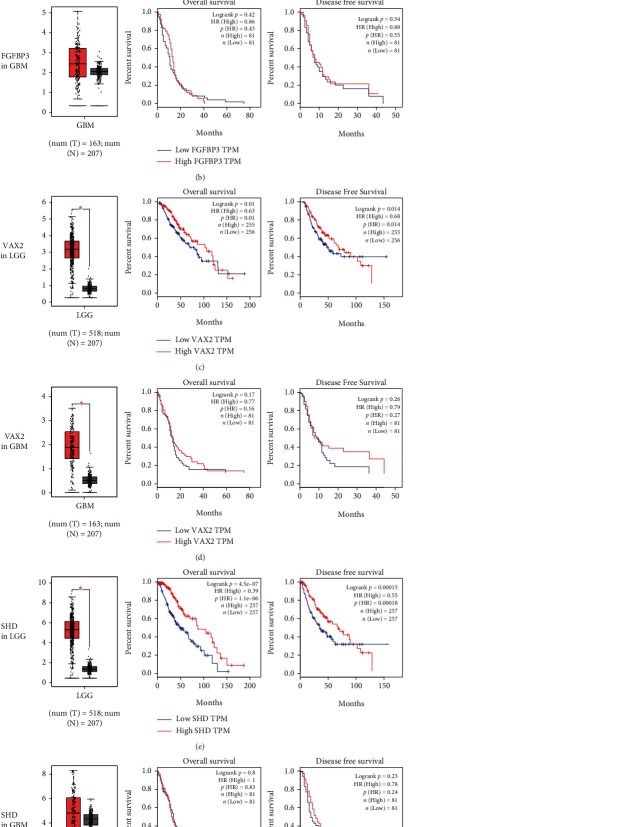
Expression and prognosis of hub genes negatively correlated with Macrophage M2. (a) Expression of FGFBP3 between LGG and normal tissue(left figure, red column: LGG, gray column: normal tissue, *p* < 0.05); the overall survival (mid figure, red curve: high FGFBP3 expression, blue curve: low FGFBP3 expression, *p* < 0.01) and disease free survival curves (right, *p* < 0.01). (b) FGFBP3 expression (*p* > 0.05), OS (*p* > 0.05), and DFS (*p* > 0.05) in GBM. (c) VAX2 expression (*p* < 0.05), OS (*p* < 0.05), and DFS (*p* < 0.05) in LGG. (d) VAX2 expression (*p* < 0.05), OS (*p* > 0.05), and DFS (*p* > 0.05) in GBM. (e) SHD expression (*p* < 0.05), OS (*p* < 0.01), and DFS (*p* < 0.01) in LGG. (f) SHD expression (*p* > 0.05) OS *p* > 0.05), and DFS prognosis (*p* > 0.05) in GBM.

Cox Proportional Hazard models for immunocytes in LGG were established by TIMER database (Table 1). Macrophages showed notable mortality risk in LGG. Hub genes negatively correlated with Macrophage M2 all showed a remarkable difference between LGG and adjacent normal tissue (Figures 5(a), 5(c), and 5(e)) while not in GBM(Figures 5(b), 5(d), and 5(f)). FGFBP3, VAX2, and SHD were all protective prognostic factors for overall survival (OS) and disease free survival (DFS) (log rank *p* < 0.05, *p*(HR) < 0.05, Figures 5(a), 5(c), and 5(e)), while no significant impact was found in GBM (log rank *p* > 0.05, *p*(HR) > 0.05, Figures 5(b), 5(d), and 5(f)).

### 3.6. Gene Set Enrichment Analysis (GSEA) of Hub Genes

**Figure 6:**
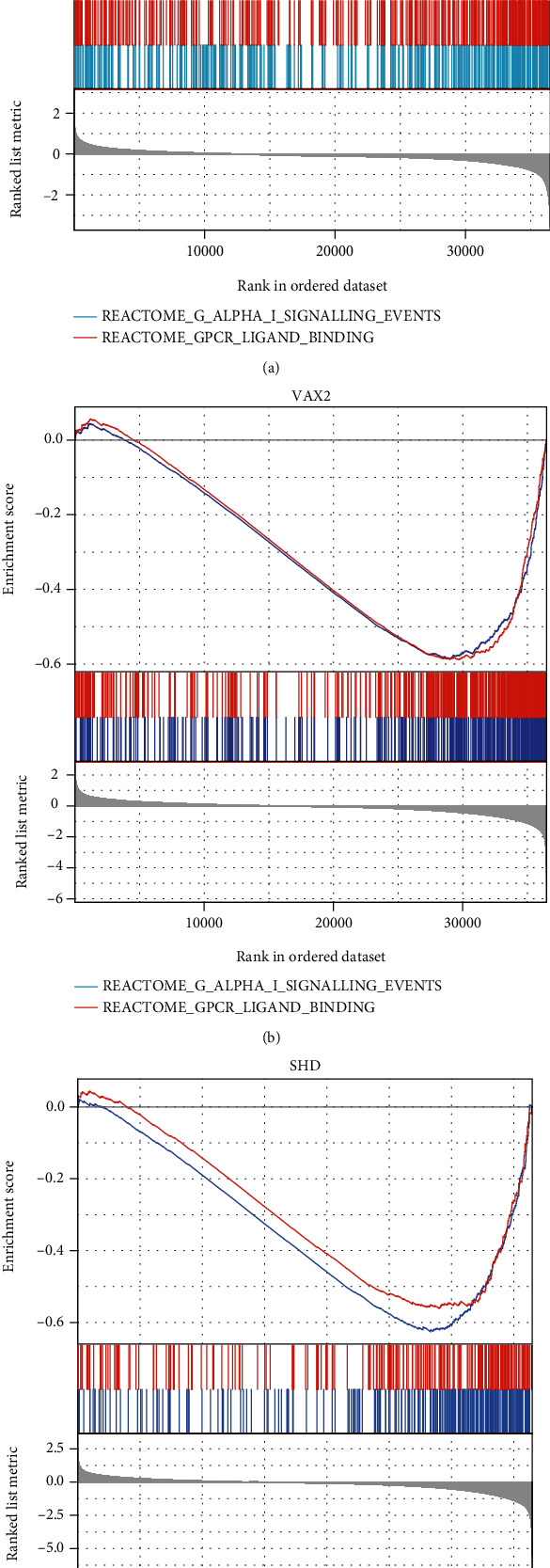
GSEA enrichment results in TCGA-LGG dataset, including FGFBP3, VAX2, and SHD.

FGFBP3, VAX2, and SHD were all enriched at the bottom of the ordered dataset with negative enrichment score peaks (Figure 6). FGFBP3 was enriched in the reactome of G alpha I signaling events with a Normalized Enrichment Score (NES) = −1.566, Nominal *p* value (NOM) *p* = 0.017, and False Discovery Rate *q* value (FDR) *q* = 0.013. FGFBP3 was also enriched in GPCR ligand binding (NES = −1.609, NOM *p* = 0.017, and FDR *q* = 0.013). VAX2 was enriched in the reactome of G alpha I signaling events (NES = −1.746, NOM *p* = 0.031, and FDR *q* = 0.023) and GPCR ligand binding (NES = −1.753, NOM *p* = 0.031, and FDR *q* = 0.023). SHD was enriched in KEGG cytokine-cytokine receptor interaction (NES = −1.669, NOM *p* = 0.027, and FDR *q* = 0.022) and NABA ECM regulators (NES = −1.494, NOM *p* = 0.027, and FDR *q* = 0.022). The results revealed that FGFBP3 and VAX2 may negatively regulated the reactome of G alpha I signaling events and GPCR ligand binding. SHD might lead to the downregulation trends of KEGG cytokine-cytokine receptor interaction and NABA ECM regulators. Moreover, other significantly enriched pathways of the above three genes were detailed in supplementary files 2.

### 3.7. Identification Independent Prognostic Risk Factors of LGG

**Figure 7:**
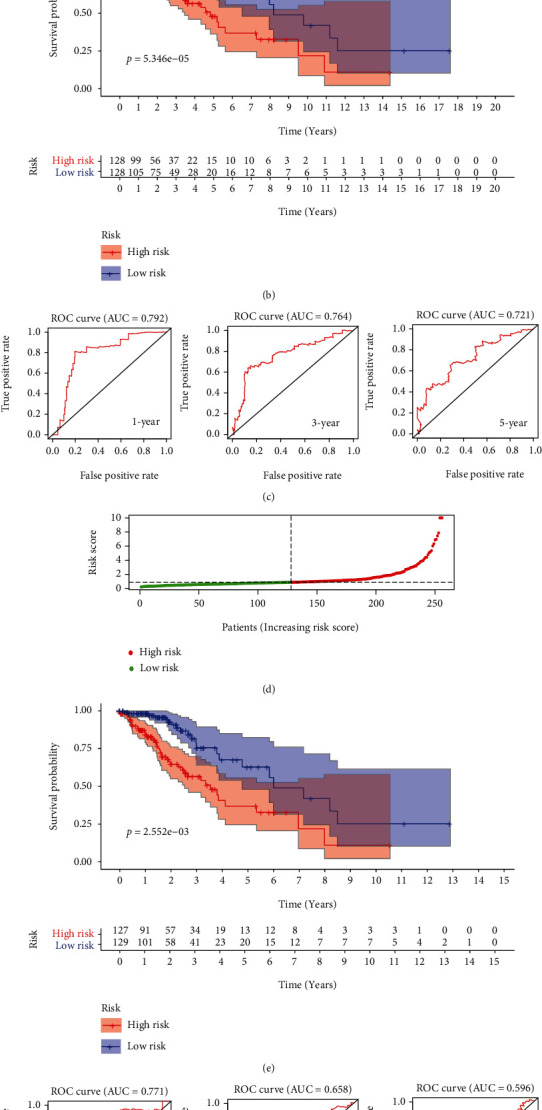
Assessment the prediction accuracy of prognostic factors. The risk score distribution in training dataset (a) and testing dataset (d). Kaplan-Meier curves for OS based on the risk score in the training dataset (b) and testing dataset (e). (shaded areas represent 95% confidence intervals. Patient number of different risk ranks at different times is listed below the curve. *p* values < 0.01). Time-dependent ROC curve of 1-year, 3-year, and 5-year survival rate in training dataset (c) and testing dataset (f).

Univariate Cox regression analysis revealed FGFBP3, VAX2, and SHD were all significantly associated with the OS of LGG in the training dataset (Table 2). Hazard Ratios (HR) of FGFBP3, VAX2, and SHD were all less than 1 with the *p* values < 0.001, indicating all of them were protective prognostic factors for LGG. After several permutation operations, the AIC value was minimized (AIC: 550.920) when only FGFBP3 and SHD were involved in the regression equation. Multivariate Cox regression showed that the HR of FGFBP3 and SHD were 0.708 and 0.734, respectively, proving that FGFBP3 and SHD were independent prognostic factors for LGG (Table 2). FGFBP3 and SHD were integrated into a predictive risk score, and the median risk score was applied as the cut-off value to distinguish high and low risk groups. In the training dataset, 128 patients were classified into the high risk group and other 128 patients were in the low risk group (Figure 7(a)). High risk group suffered from more mortality risk than the low risk group in Kaplan-Meier curve (log-rank test, Figure 7(b)). Time-dependent receiver operating characteristic (ROC) curves were drawn to evaluate the prediction accuracy. Areas under the time-dependent ROC curve (AUC) were 0.792 (1-year), 0.764(3-year), and 0.721(5-year) in the training dataset (Figure 7(c)). In the testing dataset, patients were divided into the high risk group (*n* = 127) and the low risk group (*n* = 129) (Figure 7(d)). Overall survival time of the high risk group was significantly shorter than the low risk group (Figure 7(e)). AUCs were 0.771 (1-year), 0.658 (3-year), and 0.596 (5-year) in the testing dataset (Figure 7(f)).

### 3.8. Prognostic Risk Model and Nomogram

**Figure 8:**
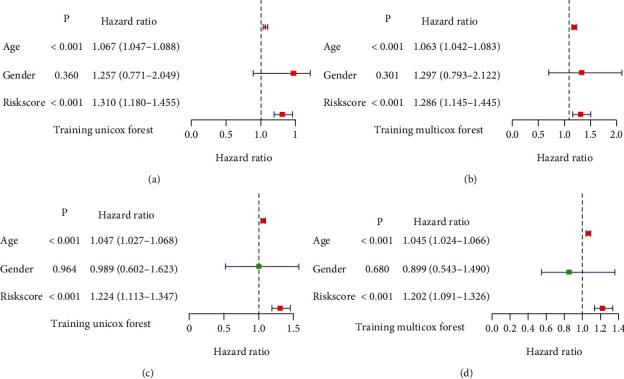
Univariate and multivariate Cox proportional hazard regression analysis of age, gender, and risk score. (a) Univariate Cox in training dataset. (b) Multivariate Cox in training dataset. (c) Univariate Cox in testing dataset. (d) Multivariate Cox in testing dataset.

Age, gender, and risk score were put into univariate and multivariate Cox proportional hazards regression analysis to select independent prognostic variables for a prognostic risk model. Univariate analysis indicated that age and risk score were significantly associated with the OS of LGG patients both in the training dataset (HR > 1, *p* < 0.001, Figure 8(a)) and the testing dataset (HR > 1, *p* < 0.001, Figure 8(c)). According to multivariate Cox regression analysis, age and risk score were independent prognostic variables in the training set (HR > 1, *p* < 0.001, Figure 8(b)) and testing set (HR > 1, *p* < 0.001, Figure 8(d)). Gender was not a prognostic factor in all the Cox analysis (*p* > 0.05). The result was consistent with the statistical analysis and clinical experience.

**Figure 9:**
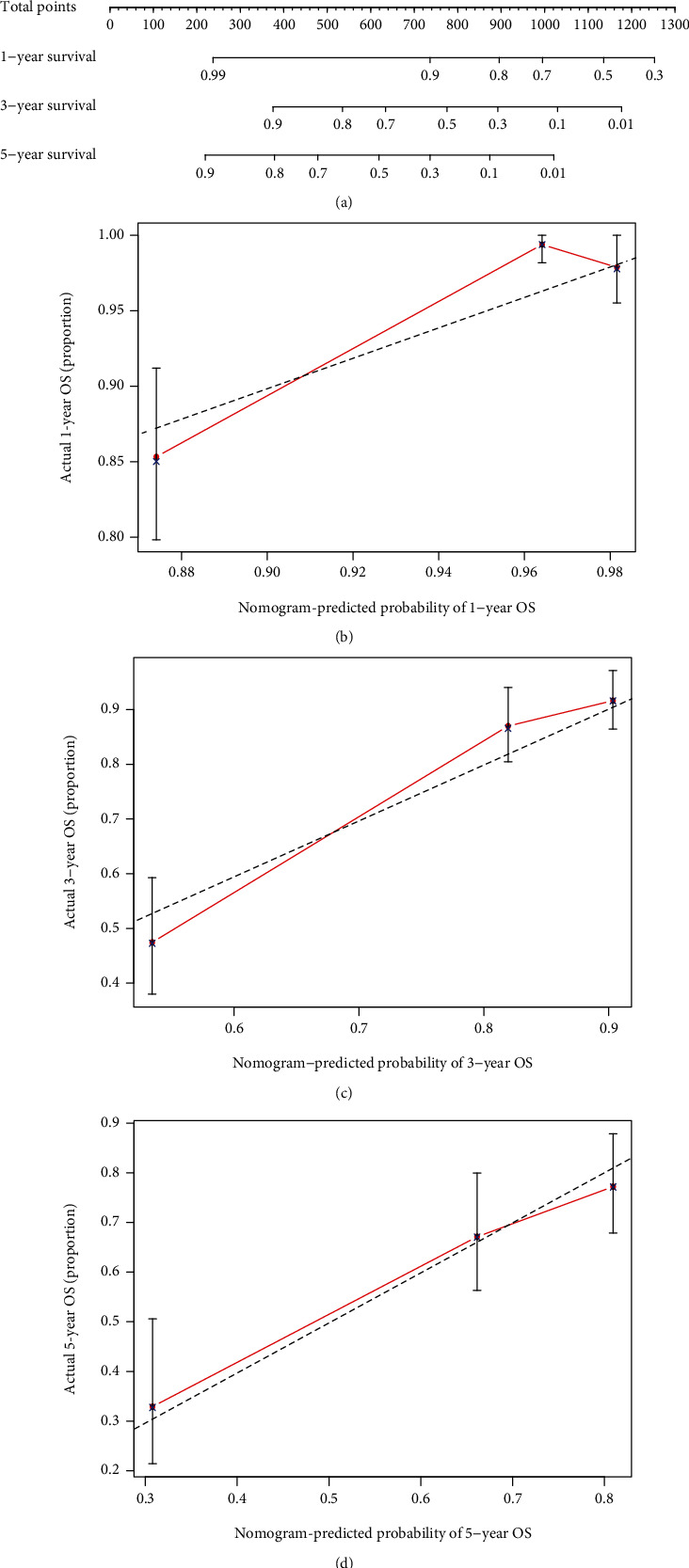
(a) Nomogram for protective prognostic factors against Macrophage M2 infiltration in LGG. (b) 1-year (c) 3-year, and (d) 5-year calibration plots of the nomogram.

**Figure 10:**
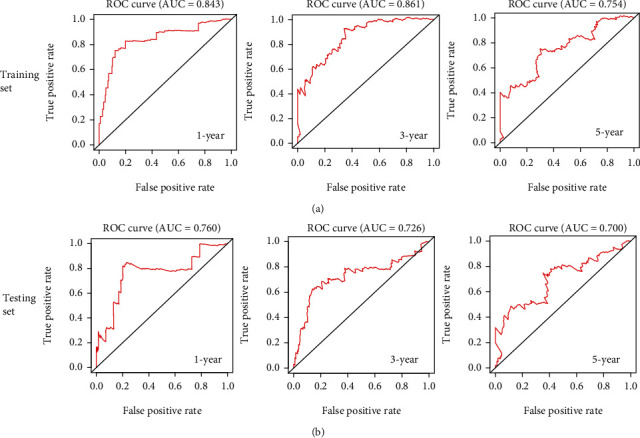
1-year, 3-year, and 5-year ROC curves for nomogram in the training dataset (a) and testing dataset (b).

A simple nomogram was designed to predict the overall survival rate based on protective prognostic genes against Macrophage M2 infiltration in low-grade glioma (Figure 9(a)). Well prediction accuracy of the nomogram was revealed by 1-year (Figure 9(b)), 3-year (Figure 9(c)), and 5-year (Figure 9(c)) calibration curves plotted in testing dataset. AUC values of ROC curves in the training set (Figure 10(a)) and testing set (Figure 10(b)) were all more than 0.7, indicating a well prediction ability in long-term survival.

## 4. Discussion

LGGs are slowly progressing tumor with ineluctable malignant potential. Biological features of LGG are quite different from GBM, and it is considered as a therapeutic time window before malignant transformation. Since LGGs often occur in adolescents and children with a life expectancy of several years, the preservation of cognitive abilities and life quality is the same vital as prolonging progression free survival [24]. Traditional surgery, chemotherapy, and radiotherapy might threaten the quality of life, while therapies for regulating tumor immune microenvironment might minimize the damage.

Macrophages M2 predominate in the immune microenvironment of glioma. They did not play the antitumor role of immune cells any longer and instead promote the occurrence, proliferation, and migration of glioma, along with immunosuppression [16, 25]. Seeking biomarkers against Macrophages M2 infiltration would be hopeful for prognosis prediction and immunotherapy.

In our study, 4 databases were chosen to search for differential genes, in which high grade glioma and samples without complete information were removed. To diminish the discrepancy of a single dataset, the overlapping differential genes were chosen for further study. Their biological functions were enriched in regulation of transsynaptic signaling, modulation of chemical synaptic transmission, ion and gated channel activity, as well as neuroactive ligand-receptor interaction. By CIBERSOR deconvolution algorithm, Macrophages M2 were found to account for the highest proportion in LGG. 43 genes negatively correlated with M2 were chosen by WGCNA analysis. Then, three hub genes which most negatively correlated with the degree of M2 immune infiltration and tumor purity were selected. FGFBP3, VAX2, and SHD were all highly expressed in LGG as protective prognostic factors for OS and DFS, while no significant difference was found in GBM. FGFBP3 and VAX2 negatively regulated the reactome of G alpha I signal events and GPCR ligand binding. SHD led to downregulation trends of KEGG cytokine-cytokine receptor interaction and NABA ECM regulators. The FGFBP3, VAX2, and SHD were all proved as protective prognostic factors. Multivariate Cox regression revealed that FGFBP3 and SHD were independent prognostic predictors. Risk scores were calculated, and a nomogram was drawn in training dataset. The prognostic predictability was verified in the testing dataset by calibration plots and ROC curves.

Fibroblast growth factor binding protein 3 (FGFBP3) is highly expressed in the central nervous system, which is essential for neuronal survival and differentiation in brain [26]. It affects carbohydrate and lipid metabolism [27] and was also found to have close relationships with breast cancer [28], angiogenesis [29], and familial pancreatic cancer [30]. The FGFBP3 regulates the FGF and FGFR signaling pathways. Duplication of the tyrosine kinase region of FGFR1 was found in 1/4 pediatric low-grade astrocytomas. cIMPACT-NOW reported that FGFR1 mutations are typical of low-grade gliomas which indicated an inert clinical behavior and long survival time [31]. The 2021 WHO classification of central nervous system defined FGFR as the Genes/Molecular Profiles Characteristic of polymorphous low-grade neuroepithelial tumor, diffuse low-grade glioma, diffuse midline glioma, and dysembryoplastic neuroepithelial tumor [6]. Ventral Anterior Homeobox 2 (VAX2) is a transcription factor that regulates the dorsoventral specification of the forebrain and Wnt signaling [32, 33]. The VAX2 has recently attracted many attentions in cancers and has been proved to regulate the malignant progression of thyroid cancer [34], breast cancer [35], and bladder cancer [36]. Src Homology 2 Domain Containing Transforming Protein (SHD) was over expressed in the cortex and frontal cortex, having a close relationship with spinal muscular atrophy type IV. In addition, with the development of nanotechnology, nanomaterials loaded with small interfering ribonucleic acid have been used in many types of cancer [37–41]. Hence, we believed that VAX2, FGFBP3, and SHD would be promoting targets for LGG therapies based on nanomaterials.

As with other studies based on the TCGA database, there were several limitations in the study. Our clinical survival study was only based on the TCGA database because the other three GEO datasets lacked integrated clinical information such as age, gender, or survival states. When extending our findings to patients of different ethnicities, caution is advised. Apart from that, the nomogram must be validated in multicenter cohorts in the future [42].

In short, FGFBP3, VAX2, and SHD might be effective prognostic predictors of LGG against Macrophage M2 infiltration. The FGFBP3 and SHD were independent predictors. Further studies were necessary for the specific mechanisms on how those genes regulating Macrophage M2 in the glioma immune microenvironment to affect LGG progression.

## 5. Conclusions

Our study identified differential genes and their biological functions between LGG and normal tissues. Various bioinformatics methods were applied, and FGFBP3, VAX2, and SHD were identified as protective prognostic factors against Macrophage M2 infiltration in LGG. The FGFBP3 and SHD were independent predictors, and a nomogram based on them could well predict the overall survival time. It might provide a new prospect for LGG immunotherapy from the perspective of regulating Macrophages M2 in central nervous system.

## Figures and Tables

**Table 1:** Cox Proportional Hazard Model of immunocytes in LGG.

Id	HR	HR.95 L	HR.95 H	*p* value
Age	1.057	1.040	1.073	0.000
B_cell	18.189	0.075	4411.806	0.300
CD8_T cell	42.057	0.054	32746.198	0.271
CD4_T cell	0.296	0.000	601.131	0.754
Macrophage	308.109	6.722	14123.299	0.003
Neutrophil	0.020	0.000	52.734	0.330
Dendritic	3.14	0.081	122.299	0.540

**Table 2:** Univariate and multivariate Cox regression results of hub genes.

*Unicox*				
Gene	HR	HR.95 L	HR.95 H	*p* value
FGFBP3	0.572	0.430	0.762	<0.001
VAX2	0.616	0.509	0.747	<0.001
SHD	0.681	0.596	0.778	<0.001
*Multicox*				
Gene	HR	HR.95 L	HR.95 H	*p* value
FGFBP3	0.708	0.526	0.953	0.023
SHD	0.734	0.636	0.849	<0.001

## Data Availability

Publicly available datasets were available from the GEO database, TCGA database, GEPIA database (http://gepia.cancer-pku.cn), and TIMER database (https://cistrome.shinyapps.io/timer/).

## Abbreviations

AUC: Achieved an area under curve
BP: Biological process
CAR: Chimeric antigen receptor
CC: Cellular component
DEGs: Differentially expressed genes
FC: Fold change
GEO: GENE EXPRESSION OMNIBUS
GEPIA: Gene Expression Profiling Interactive Analysis
GO: Gene Ontology
GSEA: Gene Set Enrichment Analysis
HR: Hazard ratio
KEGG: Kyoto Encyclopedia of Genes and Genomes
LGG: Low-grade glioma
MF: Molecular function
OS: Overall survival
ROC: Receiver Operating Characteristic Curve
TAMs: Tumor-associated macrophages
TCGA: The Cancer Genome Atlas
TIMER: Tumor Immune Estimation Resource
WGCNA: Weighted gene coexpression network analysis.

## Supplementary Materials

Supplementary Materials Supplementary file 1: clinical information of the datasets. Supplementary file 2: results of GSEA analysis. Figure S1: results of xCell, EPIC, and ssGSEA algorithms about Macrophage.
