# Supplementary material for: Protective Prognostic Biomarkers Negatively Correlated with Macrophage M2 Infiltration in Low-Grade Glioma
Source: J Oncol. 2022 Apr 8;2022:3623591. doi: 10.1155/2022/3623591 (PMC9012619; doi:10.1155/2022/3623591)
Supplement: Supplementary Materials — Supplementary file 1: clinical information of the datasets. Supplementary file 2: results of GSEA analysis. Figure S1: results of xCell, EPIC, and ssGSEA algorithms about Macrophage. [file 3623591.f1.zip › Supplement File1.docx]

| General information of the datasets | | | | |
| --- | --- | --- | --- | --- |
| Dataset | Platform | Normal | LGGs selected | DEGs |
| GSE68848 | GPL570 | 28 | 95 | 2142 |
| GSE4290 | GPL570 | 23 | 45 | 1598 |
| GSE16011 | GPL7542 | 8 | 32 | 1415 |
| TCGA-LGG | IlluminaHiSeq | 5 | 516 | 1509 |

| Clinical information of TCGA-LGG | |
| --- | --- |
| **Variables** | **Number (%)** |
| **Vital status** |  |
| Alive | 388(75.193) |
| Dead | 126(24.418) |
| Not reported | 2(0.388) |
| **Age** |  |
| ≤ 40 | 254(49.224) |
| >40 | 261(50.581) |
| Not reported | 1(0.194) |
| **Gender** |  |
| Female | 285(55.232) |
| Male | 230(44.573) |
| Not reported | 1(0.194) |
| **Histological type** |  |
| Astrocytoma | 194(37.596) |
| Oligoastrocytoma | 131(25.387) |
| Oligodendroglioma | 190(36.821) |
| Not reported | 1(0.194) |
