# Supplementary material for: Protective Prognostic Biomarkers Negatively Correlated with Macrophage M2 Infiltration in Low-Grade Glioma
Source: J Oncol. 2022 Apr 8;2022:3623591. doi: 10.1155/2022/3623591 (PMC9012619; doi:10.1155/2022/3623591)
Supplement: Supplementary Materials — Supplementary file 1: clinical information of the datasets. Supplementary file 2: results of GSEA analysis. Figure S1: results of xCell, EPIC, and ssGSEA algorithms about Macrophage. [file 3623591.f1.zip › Supplement File2.docx]

| **FGFBP3** | | | |
| --- | --- | --- | --- |
| Description | NES | p.adjust | qvalues |
| REACTOME_G_ALPHA_I_SIGNALLING_EVENTS | -1.565715421 | 0.017232747 | 0.013488324 |
| REACTOME_GPCR_LIGAND_BINDING | -1.608786 | 0.017232747 | 0.013488324 |
| REACTOME_NEUTROPHIL_DEGRANULATION | -1.965490716 | 0.017232747 | 0.013488324 |
| REACTOME_SIGNALING_BY_INTERLEUKINS | -1.909598692 | 0.017232747 | 0.013488324 |
| NABA_CORE_MATRISOME | -1.833111451 | 0.017232747 | 0.013488324 |
| REACTOME_CLASS_A_1_RHODOPSIN_LIKE_RECEPTORS_ | -1.780758873 | 0.017232747 | 0.013488324 |
| WP_IL18_SIGNALING_PATHWAY | -1.697246574 | 0.017232747 | 0.013488324 |
| REACTOME_EXTRACELLULAR_MATRIX_ORGANIZATION | -1.816934452 | 0.017232747 | 0.013488324 |
| REACTOME_LEISHMANIA_INFECTION | -2.166670405 | 0.017232747 | 0.013488324 |
| WP_FOCAL_ADHESIONPI3KAKTMTORSIGNALING_PATHWAY | -1.612478383 | 0.017232747 | 0.013488324 |
| WP_NUCLEAR_RECEPTORS_METAPATHWAY | -1.609088972 | 0.017232747 | 0.013488324 |
| WP_PI3KAKT_SIGNALING_PATHWAY | -1.566364171 | 0.017232747 | 0.013488324 |
| KEGG_CYTOKINE_CYTOKINE_RECEPTOR_INTERACTION | -2.11981501 | 0.017232747 | 0.013488324 |
| NABA_SECRETED_FACTORS | -1.873833112 | 0.017232747 | 0.013488324 |
| REACTOME_PLATELET_ACTIVATION_SIGNALING_AND_AGGREGATION | -1.765539806 | 0.017232747 | 0.013488324 |
| NABA_ECM_REGULATORS | -1.664865552 | 0.017232747 | 0.013488324 |
| KEGG_REGULATION_OF_ACTIN_CYTOSKELETON | -1.52735602 | 0.017232747 | 0.013488324 |
| NABA_ECM_GLYCOPROTEINS | -1.861880047 | 0.017232747 | 0.013488324 |
| REACTOME_ANTI_INFLAMMATORY_RESPONSE_FAVOURING_LEISHMANIA_PARASITE_INFECTION | -2.253125066 | 0.017232747 | 0.013488324 |
| REACTOME_BIOLOGICAL_OXIDATIONS | -1.529887689 | 0.017232747 | 0.013488324 |
| REACTOME_CELL_SURFACE_INTERACTIONS_AT_THE_VASCULAR_WALL | -2.458168865 | 0.017232747 | 0.013488324 |
| REACTOME_PEPTIDE_LIGAND_BINDING_RECEPTORS | -1.939516913 | 0.017232747 | 0.013488324 |
| REACTOME_FC_EPSILON_RECEPTOR_FCERI_SIGNALING | -2.395355272 | 0.017232747 | 0.013488324 |
| REACTOME_IMMUNOREGULATORY_INTERACTIONS_BETWEEN_A_LYMPHOID_AND_A_NON_LYMPHOID_CELL | -2.444287638 | 0.017232747 | 0.013488324 |
| WP_FOCAL_ADHESION | -1.608640335 | 0.017232747 | 0.013488324 |
| WP_VITAMIN_D_RECEPTOR_PATHWAY | -1.753820114 | 0.017232747 | 0.013488324 |
| KEGG_FOCAL_ADHESION | -1.645097435 | 0.017232747 | 0.013488324 |
| REACTOME_INTERFERON_SIGNALING | -1.909461923 | 0.017232747 | 0.013488324 |
| KEGG_CHEMOKINE_SIGNALING_PATHWAY | -1.855198637 | 0.017232747 | 0.013488324 |
| WP_CILIOPATHIES | -1.716044733 | 0.017232747 | 0.013488324 |
| NABA_ECM_AFFILIATED | -1.566625668 | 0.017232747 | 0.013488324 |
| REACTOME_SIGNALING_BY_THE_B_CELL_RECEPTOR_BCR_ | -2.388186401 | 0.017232747 | 0.013488324 |
| KEGG_JAK_STAT_SIGNALING_PATHWAY | -1.79442715 | 0.017232747 | 0.013488324 |
| WP_CHEMOKINE_SIGNALING_PATHWAY | -1.703919364 | 0.017232747 | 0.013488324 |
| REACTOME_FCGAMMA_RECEPTOR_FCGR_DEPENDENT_PHAGOCYTOSIS | -2.447651655 | 0.017232747 | 0.013488324 |
| REACTOME_TOLL_LIKE_RECEPTOR_CASCADES | -1.707125775 | 0.017232747 | 0.013488324 |
| PID_P53_DOWNSTREAM_PATHWAY | -1.788687879 | 0.017232747 | 0.013488324 |
| REACTOME_FCERI_MEDIATED_NF_KB_ACTIVATION | -2.521719991 | 0.017232747 | 0.013488324 |
| WP_REGULATION_OF_TOLLLIKE_RECEPTOR_SIGNALING_PATHWAY | -1.905784682 | 0.017232747 | 0.013488324 |
| KEGG_SYSTEMIC_LUPUS_ERYTHEMATOSUS | -1.884364443 | 0.017232747 | 0.013488324 |
| REACTOME_RESPONSE_TO_ELEVATED_PLATELET_CYTOSOLIC_CA2_ | -1.926392531 | 0.017232747 | 0.013488324 |
| KEGG_NATURAL_KILLER_CELL_MEDIATED_CYTOTOXICITY | -1.719903882 | 0.017232747 | 0.013488324 |
| REACTOME_REGULATION_OF_INSULIN_LIKE_GROWTH_FACTOR_IGF_TRANSPORT_AND_UPTAKE_BY_INSULIN_LIKE_GROWTH_FACTOR_BINDING_PROTEINS_IGFBPS_ | -1.865805662 | 0.017232747 | 0.013488324 |
| KEGG_CELL_ADHESION_MOLECULES_CAMS | -1.920936375 | 0.017232747 | 0.013488324 |
| WP_ADIPOGENESIS | -1.681499917 | 0.017232747 | 0.013488324 |
| REACTOME_TCR_SIGNALING | -1.768466231 | 0.017232747 | 0.013488324 |
| WP_EBOLA_VIRUS_PATHWAY_ON_HOST | -1.945152845 | 0.017232747 | 0.013488324 |
| REACTOME_ACTIVATION_OF_ANTERIOR_HOX_GENES_IN_HINDBRAIN_DEVELOPMENT_DURING_EARLY_EMBRYOGENESIS | -1.64827103 | 0.017232747 | 0.013488324 |
| KEGG_LEUKOCYTE_TRANSENDOTHELIAL_MIGRATION | -1.862039152 | 0.017232747 | 0.013488324 |
| WP_SPINAL_CORD_INJURY | -1.756728095 | 0.017232747 | 0.013488324 |
| REACTOME_COMPLEMENT_CASCADE | -2.540677504 | 0.017232747 | 0.013488324 |
| REACTOME_TNFR2_NON_CANONICAL_NF_KB_PATHWAY | -1.734375991 | 0.017232747 | 0.013488324 |
| REACTOME_INTERLEUKIN_4_AND_INTERLEUKIN_13_SIGNALING | -2.153231533 | 0.017232747 | 0.013488324 |
| REACTOME_PARASITE_INFECTION | -2.484660369 | 0.017232747 | 0.013488324 |
| KEGG_T_CELL_RECEPTOR_SIGNALING_PATHWAY | -1.782194646 | 0.017232747 | 0.013488324 |
| KEGG_TOLL_LIKE_RECEPTOR_SIGNALING_PATHWAY | -1.924945611 | 0.017232747 | 0.013488324 |
| REACTOME_ANTIGEN_PROCESSING_CROSS_PRESENTATION | -1.707062755 | 0.017232747 | 0.013488324 |
| REACTOME_COLLAGEN_FORMATION | -1.795766762 | 0.017232747 | 0.013488324 |
| WP_ALLOGRAFT_REJECTION | -2.215463821 | 0.017232747 | 0.013488324 |
| WP_SELENIUM_MICRONUTRIENT_NETWORK | -1.747357944 | 0.017232747 | 0.013488324 |
| WP_TCELL_ANTIGEN_RECEPTOR_TCR_SIGNALING_PATHWAY | -1.935729617 | 0.017232747 | 0.013488324 |
| REACTOME_BINDING_AND_UPTAKE_OF_LIGANDS_BY_SCAVENGER_RECEPTORS | -2.585070906 | 0.017232747 | 0.013488324 |
| REACTOME_FCERI_MEDIATED_MAPK_ACTIVATION | -2.534295747 | 0.017232747 | 0.013488324 |
| REACTOME_FCGR3A_MEDIATED_IL10_SYNTHESIS | -2.534513058 | 0.017232747 | 0.013488324 |
| REACTOME_INTERFERON_GAMMA_SIGNALING | -2.074762789 | 0.017232747 | 0.013488324 |
| WP_HUMAN_COMPLEMENT_SYSTEM | -2.057423569 | 0.017232747 | 0.013488324 |
| WP_SENESCENCE_AND_AUTOPHAGY_IN_CANCER | -1.783724785 | 0.017232747 | 0.013488324 |
| REACTOME_ANTIGEN_ACTIVATES_B_CELL_RECEPTOR_BCR_LEADING_TO_GENERATION_OF_SECOND_MESSENGERS | -2.490169934 | 0.017232747 | 0.013488324 |
| REACTOME_FCERI_MEDIATED_CA_2_MOBILIZATION | -2.532784383 | 0.017232747 | 0.013488324 |
| WP_TOLLLIKE_RECEPTOR_SIGNALING_PATHWAY | -1.944790898 | 0.017232747 | 0.013488324 |
| WP_VIRAL_ACUTE_MYOCARDITIS | -1.813773936 | 0.017232747 | 0.013488324 |
| KEGG_ECM_RECEPTOR_INTERACTION | -1.907944348 | 0.017232747 | 0.013488324 |
| KEGG_HEMATOPOIETIC_CELL_LINEAGE | -2.128927308 | 0.017232747 | 0.013488324 |
| REACTOME_INTEGRIN_CELL_SURFACE_INTERACTIONS | -1.925576883 | 0.017232747 | 0.013488324 |
| REACTOME_ROLE_OF_PHOSPHOLIPIDS_IN_PHAGOCYTOSIS | -2.564423314 | 0.017232747 | 0.013488324 |
| KEGG_B_CELL_RECEPTOR_SIGNALING_PATHWAY | -1.871039466 | 0.017232747 | 0.013488324 |
| KEGG_FC_EPSILON_RI_SIGNALING_PATHWAY | -1.834997026 | 0.017232747 | 0.013488324 |
| REACTOME_INITIAL_TRIGGERING_OF_COMPLEMENT | -2.571100926 | 0.017232747 | 0.013488324 |
| KEGG_COMPLEMENT_AND_COAGULATION_CASCADES | -2.016778741 | 0.017232747 | 0.013488324 |
| REACTOME_FCGR_ACTIVATION | -2.557226601 | 0.017232747 | 0.013488324 |
| REACTOME_SCAVENGING_OF_HEME_FROM_PLASMA | -2.618105403 | 0.017232747 | 0.013488324 |
| REACTOME_COSTIMULATION_BY_THE_CD28_FAMILY | -2.116328251 | 0.017232747 | 0.013488324 |
| KEGG_LEISHMANIA_INFECTION | -2.052800243 | 0.017232747 | 0.013488324 |
| KEGG_VIRAL_MYOCARDITIS | -1.902857094 | 0.017232747 | 0.013488324 |
| PID_AP1_PATHWAY | -1.995653631 | 0.017232747 | 0.013488324 |
| REACTOME_CREATION_OF_C4_AND_C2_ACTIVATORS | -2.563518613 | 0.017232747 | 0.013488324 |
| REACTOME_INTERFERON_ALPHA_BETA_SIGNALING | -2.00587957 | 0.017232747 | 0.013488324 |
| REACTOME_ROLE_OF_LAT2_NTAL_LAB_ON_CALCIUM_MOBILIZATION | -2.554772398 | 0.017232747 | 0.013488324 |
| KEGG_NOD_LIKE_RECEPTOR_SIGNALING_PATHWAY | -1.855196467 | 0.017232747 | 0.013488324 |
| PID_IL12_2PATHWAY | -2.157722582 | 0.017232747 | 0.013488324 |
| WP_TCELL_ANTIGEN_RECEPTOR_TCR_PATHWAY_DURING_STAPHYLOCOCCUS_AUREUS_INFECTION | -1.900708159 | 0.017232747 | 0.013488324 |
| PID_INTEGRIN1_PATHWAY | -1.836067104 | 0.017232747 | 0.013488324 |
| PID_CD8_TCR_DOWNSTREAM_PATHWAY | -1.997282702 | 0.017232747 | 0.013488324 |
| PID_IL4_2PATHWAY | -1.991581601 | 0.017232747 | 0.013488324 |
| REACTOME_ASSEMBLY_OF_COLLAGEN_FIBRILS_AND_OTHER_MULTIMERIC_STRUCTURES | -1.846342694 | 0.017232747 | 0.013488324 |
| REACTOME_CD22_MEDIATED_BCR_REGULATION | -2.569038204 | 0.017232747 | 0.013488324 |
| WP_HEMATOPOIETIC_STEM_CELL_DIFFERENTIATION | -1.864414859 | 0.017232747 | 0.013488324 |
| WP_TYROBP_CAUSAL_NETWORK | -2.289829796 | 0.017232747 | 0.013488324 |
| PID_BCR_5PATHWAY | -1.794841864 | 0.017232747 | 0.013488324 |
| PID_TCR_PATHWAY | -1.955580765 | 0.017232747 | 0.013488324 |
| WP_LUNG_FIBROSIS | -1.97873958 | 0.017232747 | 0.013488324 |
| PID_FCER1_PATHWAY | -1.791723084 | 0.017232747 | 0.013488324 |
| WP_COMPLEMENT_AND_COAGULATION_CASCADES | -1.983361541 | 0.017232747 | 0.013488324 |
| REACTOME_CHEMOKINE_RECEPTORS_BIND_CHEMOKINES | -2.146403668 | 0.017232747 | 0.013488324 |
| REACTOME_NON_INTEGRIN_MEMBRANE_ECM_INTERACTIONS | -1.834686247 | 0.017232747 | 0.013488324 |
| KEGG_AUTOIMMUNE_THYROID_DISEASE | -2.114610664 | 0.017232747 | 0.013488324 |
| PID_CD8_TCR_PATHWAY | -1.918539961 | 0.017232747 | 0.013488324 |
| KEGG_INTESTINAL_IMMUNE_NETWORK_FOR_IGA_PRODUCTION | -2.057909687 | 0.017232747 | 0.013488324 |
| PID_NFAT_TFPATHWAY | -1.959019726 | 0.017232747 | 0.013488324 |
| REACTOME_ELASTIC_FIBRE_FORMATION | -1.883772169 | 0.017232747 | 0.013488324 |
| REACTOME_INTERLEUKIN_10_SIGNALING | -2.244243778 | 0.017232747 | 0.013488324 |
| KEGG_TYROSINE_METABOLISM | -1.808297393 | 0.017232747 | 0.013488324 |
| PID_UPA_UPAR_PATHWAY | -2.066418655 | 0.017232747 | 0.013488324 |
| PID_INTEGRIN3_PATHWAY | -1.90529793 | 0.017232747 | 0.013488324 |
| KEGG_TYPE_I_DIABETES_MELLITUS | -1.958462577 | 0.017232747 | 0.013488324 |
| WP_FIBRIN_COMPLEMENT_RECEPTOR_3_SIGNALING_PATHWAY | -1.84017684 | 0.017232747 | 0.013488324 |
| WP_NUCLEOTIDEBINDING_OLIGOMERIZATION_DOMAIN_NOD_PATHWAY | -1.824369053 | 0.017232747 | 0.013488324 |
| REACTOME_DAP12_INTERACTIONS | -1.848681249 | 0.017232747 | 0.013488324 |
| WP_MICROGLIA_PATHOGEN_PHAGOCYTOSIS_PATHWAY | -2.185658463 | 0.017232747 | 0.013488324 |
| BIOCARTA_FCER1_PATHWAY | -1.841760599 | 0.017232747 | 0.013488324 |
| WP_INTERACTIONS_BETWEEN_IMMUNE_CELLS_AND_MICRORNAS_IN_TUMOR_MICROENVIRONMENT | -1.890548441 | 0.017232747 | 0.013488324 |
| WP_MIRNA_TARGETS_IN_ECM_AND_MEMBRANE_RECEPTORS | -1.782004958 | 0.017232747 | 0.013488324 |
| REACTOME_MOLECULES_ASSOCIATED_WITH_ELASTIC_FIBRES | -1.794710226 | 0.017232747 | 0.013488324 |
| REACTOME_SMOOTH_MUSCLE_CONTRACTION | -1.735317238 | 0.017232747 | 0.013488324 |
| BIOCARTA_IL2RB_PATHWAY | -1.837679119 | 0.017232747 | 0.013488324 |
| KEGG_GRAFT_VERSUS_HOST_DISEASE | -2.094890828 | 0.017232747 | 0.013488324 |
| PID_FRA_PATHWAY | -1.936545381 | 0.017232747 | 0.013488324 |
| PID_IL23_PATHWAY | -1.829269761 | 0.017232747 | 0.013488324 |
| REACTOME_GENERATION_OF_SECOND_MESSENGER_MOLECULES | -2.114189479 | 0.017232747 | 0.013488324 |
| WP_TYPE_II_INTERFERON_SIGNALING_IFNG | -1.856101473 | 0.017232747 | 0.013488324 |
| SIG_PIP3_SIGNALING_IN_B_LYMPHOCYTES | -1.764111861 | 0.017232747 | 0.013488324 |
| KEGG_ALLOGRAFT_REJECTION | -2.142297504 | 0.017232747 | 0.013488324 |
| KEGG_PRIMARY_IMMUNODEFICIENCY | -1.935852182 | 0.017232747 | 0.013488324 |
| REACTOME_GPVI_MEDIATED_ACTIVATION_CASCADE | -1.895021612 | 0.017232747 | 0.013488324 |
| WP_PHOTODYNAMIC_THERAPYINDUCED_NFKB_SURVIVAL_SIGNALING | -1.844406068 | 0.017232747 | 0.013488324 |
| PID_IL12_STAT4_PATHWAY | -1.79044712 | 0.017232747 | 0.013488324 |
| WP_INFLAMMATORY_RESPONSE_PATHWAY | -2.032766108 | 0.017232747 | 0.013488324 |
| KEGG_LINOLEIC_ACID_METABOLISM | -1.765546388 | 0.017232747 | 0.013488324 |
| REACTOME_ACYL_CHAIN_REMODELLING_OF_PE | -1.818407247 | 0.017232747 | 0.013488324 |
| WP_SELECTIVE_EXPRESSION_OF_CHEMOKINE_RECEPTORS_DURING_TCELL_POLARIZATION | -1.960716005 | 0.017232747 | 0.013488324 |
| KEGG_ASTHMA | -2.057606947 | 0.017232747 | 0.013488324 |
| PID_INTEGRIN2_PATHWAY | -1.882317884 | 0.017232747 | 0.013488324 |
| REACTOME_TNFS_BIND_THEIR_PHYSIOLOGICAL_RECEPTORS | -1.873762817 | 0.017232747 | 0.013488324 |
| BIOCARTA_INFLAM_PATHWAY | -1.968247848 | 0.017232747 | 0.013488324 |
| REACTOME_SYNDECAN_INTERACTIONS | -1.960324481 | 0.017232747 | 0.013488324 |
| WP_HYPOTHESIZED_PATHWAYS_IN_PATHOGENESIS_OF_CARDIOVASCULAR_DISEASE | -1.819392364 | 0.017232747 | 0.013488324 |
| BIOCARTA_NKT_PATHWAY | -1.939740007 | 0.017232747 | 0.013488324 |
| PID_INTEGRIN_CS_PATHWAY | -1.835337445 | 0.017232747 | 0.013488324 |
| REACTOME_PD_1_SIGNALING | -2.263675996 | 0.017232747 | 0.013488324 |
| WP_CYTOKINES_AND_INFLAMMATORY_RESPONSE | -1.913715426 | 0.017232747 | 0.013488324 |
| REACTOME_GROWTH_HORMONE_RECEPTOR_SIGNALING | -1.802167175 | 0.017232747 | 0.013488324 |
| WP_IL1_AND_MEGAKARYOCYTES_IN_OBESITY | -1.905913694 | 0.017232747 | 0.013488324 |
| WP_CANCER_IMMUNOTHERAPY_BY_PD1_BLOCKADE | -1.99259897 | 0.017232747 | 0.013488324 |
| WP_COMPLEMENT_ACTIVATION | -1.947247422 | 0.017232747 | 0.013488324 |
| BIOCARTA_TH1TH2_PATHWAY | -1.841528373 | 0.017232747 | 0.013488324 |
| WP_PATHOGENESIS_OF_SARSCOV2_MEDIATED_BY_NSP9NSP10_COMPLEX | -1.997652016 | 0.017232747 | 0.013488324 |
| BIOCARTA_CSK_PATHWAY | -1.910616041 | 0.017232747 | 0.013488324 |
| BIOCARTA_CTLA4_PATHWAY | -1.986765054 | 0.017232747 | 0.013488324 |
| BIOCARTA_COMP_PATHWAY | -2.02845303 | 0.017232747 | 0.013488324 |
| BIOCARTA_TOB1_PATHWAY | -1.857784723 | 0.017232747 | 0.013488324 |
| REACTOME_REGULATION_OF_TLR_BY_ENDOGENOUS_LIGAND | -1.787439818 | 0.017232747 | 0.013488324 |
| BIOCARTA_DC_PATHWAY | -1.854308925 | 0.017232747 | 0.013488324 |
| BIOCARTA_LAIR_PATHWAY | -1.872090226 | 0.017232747 | 0.013488324 |
| REACTOME_ACYL_CHAIN_REMODELLING_OF_PI | -1.773640754 | 0.017232747 | 0.013488324 |
| REACTOME_TNF_RECEPTOR_SUPERFAMILY_TNFSF_MEMBERS_MEDIATING_NON_CANONICAL_NF_KB_PATHWAY | -1.773159677 | 0.017232747 | 0.013488324 |
| WP_CELLS_AND_MOLECULES_INVOLVED_IN_LOCAL_ACUTE_INFLAMMATORY_RESPONSE | -1.872090226 | 0.017232747 | 0.013488324 |
| WP_PLATELETMEDIATED_INTERACTIONS_WITH_VASCULAR_AND_CIRCULATING_CELLS | -1.955394954 | 0.017232747 | 0.013488324 |
| BIOCARTA_GRANULOCYTES_PATHWAY | -1.910215038 | 0.017232747 | 0.013488324 |
| BIOCARTA_IL17_PATHWAY | -1.849391133 | 0.017232747 | 0.013488324 |
| BIOCARTA_NO2IL12_PATHWAY | -1.818801125 | 0.017232747 | 0.013488324 |
| SA_MMP_CYTOKINE_CONNECTION | -1.767308887 | 0.017232747 | 0.013488324 |
| WP_COVID19_ADVERSE_OUTCOME_PATHWAY | -1.811082362 | 0.017232747 | 0.013488324 |
| BIOCARTA_ASBCELL_PATHWAY | -1.823955701 | 0.017232747 | 0.013488324 |
| BIOCARTA_CLASSIC_PATHWAY | -1.925344755 | 0.017232747 | 0.013488324 |
| BIOCARTA_CTL_PATHWAY | -1.878102856 | 0.017232747 | 0.013488324 |
| BIOCARTA_IL10_PATHWAY | -1.712883568 | 0.017232747 | 0.013488324 |
| WP_CANCER_IMMUNOTHERAPY_BY_CTLA4_BLOCKADE | -1.738014223 | 0.017232747 | 0.013488324 |
| BIOCARTA_BLYMPHOCYTE_PATHWAY | -1.904480001 | 0.017232747 | 0.013488324 |
| BIOCARTA_LECTIN_PATHWAY | -1.783086479 | 0.017232747 | 0.013488324 |
| BIOCARTA_TCRA_PATHWAY | -1.955707059 | 0.017232747 | 0.013488324 |
| BIOCARTA_TCYTOTOXIC_PATHWAY | -1.956958312 | 0.017232747 | 0.013488324 |
| BIOCARTA_THELPER_PATHWAY | -1.956903785 | 0.017232747 | 0.013488324 |
| REACTOME_INTERLEUKIN_2_SIGNALING | -1.730605114 | 0.017232747 | 0.013488324 |
| BIOCARTA_IL5_PATHWAY | -1.712548262 | 0.017232747 | 0.013488324 |
| BIOCARTA_MONOCYTE_PATHWAY | -1.734168396 | 0.017232747 | 0.013488324 |
| REACTOME_ENDOSOMAL_VACUOLAR_PATHWAY | -1.756763416 | 0.017232747 | 0.013488324 |
| REACTOME_METALLOTHIONEINS_BIND_METALS | -1.707181409 | 0.017232747 | 0.013488324 |
| WP_VEGFAVEGFR2_SIGNALING_PATHWAY | -1.398489493 | 0.025150905 | 0.019685983 |
| KEGG_PATHWAYS_IN_CANCER | -1.436104256 | 0.025150905 | 0.019685983 |
| REACTOME_G_ALPHA_Q_SIGNALLING_EVENTS | -1.523511875 | 0.025150905 | 0.019685983 |
| REACTOME_DEGRADATION_OF_THE_EXTRACELLULAR_MATRIX | -1.585911616 | 0.025376643 | 0.019862671 |
| PID_CXCR4_PATHWAY | -1.642441636 | 0.025533516 | 0.019985458 |
| REACTOME_VISUAL_PHOTOTRANSDUCTION | -1.635921448 | 0.025533516 | 0.019985458 |
| REACTOME_ANTIMICROBIAL_PEPTIDES | -1.677856414 | 0.025533516 | 0.019985458 |
| WP_HAIR_FOLLICLE_DEVELOPMENT_CYTODIFFERENTIATION_PART_3_OF_3 | -1.658341134 | 0.025533516 | 0.019985458 |
| KEGG_ANTIGEN_PROCESSING_AND_PRESENTATION | -1.726416201 | 0.025537085 | 0.019988252 |
| REACTOME_ECM_PROTEOGLYCANS | -1.733147685 | 0.025542029 | 0.019992121 |
| KEGG_METABOLISM_OF_XENOBIOTICS_BY_CYTOCHROME_P450 | -1.638589019 | 0.025685117 | 0.020104118 |
| REACTOME_COLLAGEN_DEGRADATION | -1.670223626 | 0.025776832 | 0.020175905 |
| PID_IL2_1PATHWAY | -1.794686664 | 0.026214128 | 0.020518183 |
| WP_PROSTAGLANDIN_SYNTHESIS_AND_REGULATION | -1.768575285 | 0.026522829 | 0.020759808 |
| WP_IL2_SIGNALING_PATHWAY | -1.734817391 | 0.026559608 | 0.020788595 |
| BIOCARTA_TCR_PATHWAY | -1.797701976 | 0.026559608 | 0.020788595 |
| WP_ZINC_HOMEOSTASIS | -1.71340812 | 0.027137895 | 0.021241229 |
| REACTOME_LAMININ_INTERACTIONS | -1.731068291 | 0.027223439 | 0.021308185 |
| REACTOME_SURFACTANT_METABOLISM | -1.738706198 | 0.027223439 | 0.021308185 |
| PID_TCR_CALCIUM_PATHWAY | -1.764970646 | 0.027646819 | 0.021639571 |
| REACTOME_THE_CANONICAL_RETINOID_CYCLE_IN_RODS_TWILIGHT_VISION_ | -1.745736335 | 0.028232637 | 0.022098099 |
| BIOCARTA_IL2_PATHWAY | -1.762863349 | 0.028232637 | 0.022098099 |
| WP_LTF_DANGER_SIGNAL_RESPONSE_PATHWAY | -1.766694711 | 0.028604636 | 0.022389268 |
| WP_OVERVIEW_OF_NANOPARTICLE_EFFECTS | -1.770506394 | 0.028604636 | 0.022389268 |
| REACTOME_ACYL_CHAIN_REMODELLING_OF_PG | -1.762146917 | 0.028799049 | 0.022541439 |
| KEGG_MAPK_SIGNALING_PATHWAY | -1.429753667 | 0.033361115 | 0.026112234 |
| WP_GPCRS_CLASS_A_RHODOPSINLIKE | -1.459171217 | 0.033361115 | 0.026112234 |
| REACTOME_TOLL_LIKE_RECEPTOR_4_TLR4_CASCADE | -1.572797859 | 0.034041394 | 0.026644699 |
| KEGG_LYSOSOME | -1.561075687 | 0.034041394 | 0.026644699 |
| KEGG_FC_GAMMA_R_MEDIATED_PHAGOCYTOSIS | -1.644475939 | 0.034525869 | 0.027023905 |
| WP_CYTOSOLIC_DNASENSING_PATHWAY | -1.663945506 | 0.034666472 | 0.027133957 |
| WP_PRIMARY_FOCAL_SEGMENTAL_GLOMERULOSCLEROSIS_FSGS | -1.648415102 | 0.034666472 | 0.027133957 |
| WP_FOLATE_METABOLISM | -1.718367436 | 0.034666472 | 0.027133957 |
| WP_NONGENOMIC_ACTIONS_OF_125_DIHYDROXYVITAMIN_D3 | -1.74369241 | 0.034666472 | 0.027133957 |
| PID_MYC_REPRESS_PATHWAY | -1.682380878 | 0.034696859 | 0.027157741 |
| KEGG_ARACHIDONIC_ACID_METABOLISM | -1.75805127 | 0.034728992 | 0.027182892 |
| WP_IL4_SIGNALING_PATHWAY | -1.762360383 | 0.035069499 | 0.027449412 |
| REACTOME_INTERLEUKIN_3_INTERLEUKIN_5_AND_GM_CSF_SIGNALING | -1.783550483 | 0.03518588 | 0.027540505 |
| PID_DELTA_NP63_PATHWAY | -1.753369664 | 0.03518588 | 0.027540505 |
| WP_TNF_RELATED_WEAK_INDUCER_OF_APOPTOSIS_TWEAK_SIGNALING_PATHWAY | -1.685556012 | 0.035466288 | 0.027759985 |
| REACTOME_INTERLEUKIN_2_FAMILY_SIGNALING | -1.755307196 | 0.035471056 | 0.027763716 |
| REACTOME_DETOXIFICATION_OF_REACTIVE_OXYGEN_SPECIES | -1.674427034 | 0.036262864 | 0.028383476 |
| PID_LYMPH_ANGIOGENESIS_PATHWAY | -1.771160235 | 0.037917088 | 0.029678261 |
| REACTOME_RHO_GTPASES_ACTIVATE_NADPH_OXIDASES | -1.777499908 | 0.037985414 | 0.029731741 |
| REACTOME_PRESYNAPTIC_DEPOLARIZATION_AND_CALCIUM_CHANNEL_OPENING | 2.173652436 | 0.038630449 | 0.03023662 |
| WP_MIR5093P_ALTERATION_OF_YAP1ECM_AXIS | -1.760291605 | 0.03930514 | 0.03076471 |
| KEGG_TERPENOID_BACKBONE_BIOSYNTHESIS | 2.248702351 | 0.041287833 | 0.032316593 |
| WP_CHOLESTEROL_BIOSYNTHESIS_PATHWAY | 2.684602155 | 0.041287833 | 0.032316593 |
| REACTOME_NEGATIVE_REGULATION_OF_THE_PI3K_AKT_NETWORK | -1.510925638 | 0.041287833 | 0.032316593 |
| WP_INTEGRINMEDIATED_CELL_ADHESION | -1.567539972 | 0.041287833 | 0.032316593 |
| REACTOME_STIMULI_SENSING_CHANNELS | -1.573378832 | 0.041287833 | 0.032316593 |
| WP_B_CELL_RECEPTOR_SIGNALING_PATHWAY | -1.55456859 | 0.041287833 | 0.032316593 |
| WP_APOPTOSIS | -1.570948351 | 0.041287833 | 0.032316593 |
| PID_REG_GR_PATHWAY | -1.652592567 | 0.041287833 | 0.032316593 |
| PID_SMAD2_3NUCLEAR_PATHWAY | -1.613230086 | 0.041287833 | 0.032316593 |
| REACTOME_CONSTITUTIVE_SIGNALING_BY_ABERRANT_PI3K_IN_CANCER | -1.641302676 | 0.041287833 | 0.032316593 |
| WP_PEPTIDE_GPCRS | -1.608843168 | 0.041287833 | 0.032316593 |
| WP_REGULATORY_CIRCUITS_OF_THE_STAT3_SIGNALING_PATHWAY | -1.683148315 | 0.041382615 | 0.03239078 |
| PID_HIF1_TFPATHWAY | -1.646138127 | 0.041416713 | 0.032417469 |
| PID_SHP2_PATHWAY | -1.64967633 | 0.041416713 | 0.032417469 |
| KEGG_STEROID_BIOSYNTHESIS | 1.951658135 | 0.041416713 | 0.032417469 |
| KEGG_CYTOSOLIC_DNA_SENSING_PATHWAY | -1.683573671 | 0.041416713 | 0.032417469 |
| PID_FGF_PATHWAY | -1.662916856 | 0.041416713 | 0.032417469 |
| WP_VITAMIN_B12_METABOLISM | -1.67521457 | 0.041416713 | 0.032417469 |
| WP_IL3_SIGNALING_PATHWAY | -1.735497777 | 0.041416713 | 0.032417469 |
| REACTOME_METABOLISM_OF_FAT_SOLUBLE_VITAMINS | -1.683089278 | 0.041416713 | 0.032417469 |
| PID_IL6_7_PATHWAY | -1.732155905 | 0.041416713 | 0.032417469 |
| REACTOME_SEROTONIN_NEUROTRANSMITTER_RELEASE_CYCLE | 1.952925811 | 0.041416713 | 0.032417469 |
| WP_SREBF_AND_MIR33_IN_CHOLESTEROL_AND_LIPID_HOMEOSTASIS | 2.055962866 | 0.041416713 | 0.032417469 |
| WP_NEPHROTIC_SYNDROME | -1.71913512 | 0.041487071 | 0.03247254 |
| WP_GENES_CONTROLLING_RENAL_NEPHROGENESIS | -1.675222337 | 0.042422456 | 0.033204679 |
| REACTOME_GABA_SYNTHESIS_RELEASE_REUPTAKE_AND_DEGRADATION | 1.765008638 | 0.042422456 | 0.033204679 |
| REACTOME_NA_CL_DEPENDENT_NEUROTRANSMITTER_TRANSPORTERS | 2.115445856 | 0.042422456 | 0.033204679 |
| ST_ADRENERGIC | -1.6618146 | 0.042422456 | 0.033204679 |
| REACTOME_INTERLEUKIN_7_SIGNALING | -1.720883288 | 0.04254614 | 0.033301489 |
| PID_SYNDECAN_4_PATHWAY | -1.729834237 | 0.04254614 | 0.033301489 |
| REACTOME_RECEPTOR_TYPE_TYROSINE_PROTEIN_PHOSPHATASES | 1.915745587 | 0.043044077 | 0.033691231 |
| REACTOME_ACYL_CHAIN_REMODELLING_OF_PC | -1.705832923 | 0.043237634 | 0.033842731 |
| REACTOME_INTERLEUKIN_RECEPTOR_SHC_SIGNALING | -1.695899829 | 0.043237634 | 0.033842731 |
| PID_IL27_PATHWAY | -1.726468464 | 0.043710718 | 0.034213021 |
| WP_NRF2_PATHWAY | -1.499385723 | 0.045045899 | 0.035258087 |
| REACTOME_DOPAMINE_NEUROTRANSMITTER_RELEASE_CYCLE | 1.905202703 | 0.045045899 | 0.035258087 |
| REACTOME_GLUTAMATE_NEUROTRANSMITTER_RELEASE_CYCLE | 1.777148996 | 0.046296296 | 0.036236791 |
| PID_AVB3_INTEGRIN_PATHWAY | -1.57436006 | 0.046459993 | 0.036364919 |
| REACTOME_RESPONSE_TO_METAL_IONS | -1.659405885 | 0.046459993 | 0.036364919 |
| WP_TRANSCRIPTIONAL_CASCADE_REGULATING_ADIPOGENESIS | -1.68199521 | 0.046459993 | 0.036364919 |
| KEGG_DRUG_METABOLISM_CYTOCHROME_P450 | -1.585927926 | 0.046601523 | 0.036475696 |
| REACTOME_CHOLESTEROL_BIOSYNTHESIS | 2.916245081 | 0.046683047 | 0.036539506 |
| REACTOME_INTERLEUKIN_12_FAMILY_SIGNALING | -1.623840324 | 0.047073701 | 0.036845277 |
| WP_COPPER_HOMEOSTASIS | -1.632204946 | 0.047147712 | 0.036903207 |
| WP_TGFBETA_RECEPTOR_SIGNALING | -1.629362437 | 0.047147712 | 0.036903207 |
| PID_SYNDECAN_1_PATHWAY | -1.698671164 | 0.047492401 | 0.037173 |
| SIG_BCR_SIGNALING_PATHWAY | -1.609800548 | 0.047492401 | 0.037173 |
| WP_PHOTODYNAMIC_THERAPYINDUCED_HIF1_SURVIVAL_SIGNALING | -1.652285404 | 0.04925383 | 0.038551696 |
| KEGG_PRION_DISEASES | -1.675186786 | 0.049424799 | 0.038685516 |

| **SHD** | | | |
| --- | --- | --- | --- |
| Description | NES | p.adjust | qvalues |
| KEGG_CYTOKINE_CYTOKINE_RECEPTOR_INTERACTION | -1.669317558 | 0.027168629 | 0.022132 |
| NABA_ECM_REGULATORS | -1.493844775 | 0.027168629 | 0.022132 |
| NABA_SECRETED_FACTORS | -1.492257567 | 0.027168629 | 0.022132 |
| REACTOME_CLASS_A_1_RHODOPSIN_LIKE_RECEPTORS_ | -1.741221016 | 0.027168629 | 0.022132 |
| REACTOME_EXTRACELLULAR_MATRIX_ORGANIZATION | -1.620376836 | 0.027168629 | 0.022132 |
| REACTOME_G_ALPHA_I_SIGNALLING_EVENTS | -1.575195015 | 0.027168629 | 0.022132 |
| REACTOME_GPCR_LIGAND_BINDING | -1.655276822 | 0.027168629 | 0.022132 |
| REACTOME_LEISHMANIA_INFECTION | -1.670989388 | 0.027168629 | 0.022132 |
| REACTOME_NEURONAL_SYSTEM | -1.338280937 | 0.027168629 | 0.022132 |
| REACTOME_NEUTROPHIL_DEGRANULATION | -1.563455023 | 0.027168629 | 0.022132 |
| REACTOME_SIGNALING_BY_INTERLEUKINS | -1.534724555 | 0.027168629 | 0.022132 |
| WP_FOCAL_ADHESIONPI3KAKTMTORSIGNALING_PATHWAY | -1.435495461 | 0.027168629 | 0.022132 |
| WP_GPCRS_CLASS_A_RHODOPSINLIKE | -1.672605338 | 0.027168629 | 0.022132 |
| WP_NUCLEAR_RECEPTORS_METAPATHWAY | -1.49058952 | 0.027168629 | 0.022132 |
| KEGG_MAPK_SIGNALING_PATHWAY | -1.459262676 | 0.027168629 | 0.022132 |
| KEGG_NEUROACTIVE_LIGAND_RECEPTOR_INTERACTION | -1.582783146 | 0.027168629 | 0.022132 |
| NABA_CORE_MATRISOME | -1.737341706 | 0.027168629 | 0.022132 |
| REACTOME_ANTI_INFLAMMATORY_RESPONSE_FAVOURING_LEISHMANIA_PARASITE_INFECTION | -1.770227402 | 0.027168629 | 0.022132 |
| REACTOME_PLATELET_ACTIVATION_SIGNALING_AND_AGGREGATION | -1.511561159 | 0.027168629 | 0.022132 |
| WP_MAPK_SIGNALING_PATHWAY | -1.422753361 | 0.027168629 | 0.022132 |
| KEGG_REGULATION_OF_ACTIN_CYTOSKELETON | -1.466367218 | 0.027168629 | 0.022132 |
| REACTOME_FCGAMMA_RECEPTOR_FCGR_DEPENDENT_PHAGOCYTOSIS | -1.87999383 | 0.027168629 | 0.022132 |
| REACTOME_G_ALPHA_Q_SIGNALLING_EVENTS | -1.571178107 | 0.027168629 | 0.022132 |
| KEGG_CALCIUM_SIGNALING_PATHWAY | -1.557263217 | 0.027168629 | 0.022132 |
| KEGG_JAK_STAT_SIGNALING_PATHWAY | -1.588547893 | 0.027168629 | 0.022132 |
| NABA_ECM_AFFILIATED | -1.504280023 | 0.027168629 | 0.022132 |
| REACTOME_FC_EPSILON_RECEPTOR_FCERI_SIGNALING | -1.732294779 | 0.027168629 | 0.022132 |
| REACTOME_IMMUNOREGULATORY_INTERACTIONS_BETWEEN_A_LYMPHOID_AND_A_NON_LYMPHOID_CELL | -1.917264754 | 0.027168629 | 0.022132 |
| REACTOME_SIGNALING_BY_THE_B_CELL_RECEPTOR_BCR_ | -1.765866385 | 0.027168629 | 0.022132 |
| WP_MYOMETRIAL_RELAXATION_AND_CONTRACTION_PATHWAYS | -1.591645718 | 0.027168629 | 0.022132 |
| WP_VITAMIN_D_RECEPTOR_PATHWAY | -1.536888644 | 0.027168629 | 0.022132 |
| KEGG_CELL_ADHESION_MOLECULES_CAMS | -1.638565257 | 0.027168629 | 0.022132 |
| KEGG_FOCAL_ADHESION | -1.542972778 | 0.027168629 | 0.022132 |
| KEGG_NATURAL_KILLER_CELL_MEDIATED_CYTOTOXICITY | -1.586209785 | 0.027168629 | 0.022132 |
| NABA_ECM_GLYCOPROTEINS | -1.716851696 | 0.027168629 | 0.022132 |
| REACTOME_CELL_SURFACE_INTERACTIONS_AT_THE_VASCULAR_WALL | -1.885925188 | 0.027168629 | 0.022132 |
| REACTOME_FCERI_MEDIATED_NF_KB_ACTIVATION | -1.952785507 | 0.027168629 | 0.022132 |
| REACTOME_INTERFERON_SIGNALING | -1.594599782 | 0.027168629 | 0.022132 |
| REACTOME_PEPTIDE_LIGAND_BINDING_RECEPTORS | -1.77820606 | 0.027168629 | 0.022132 |
| REACTOME_RESPONSE_TO_ELEVATED_PLATELET_CYTOSOLIC_CA2_ | -1.666216809 | 0.027168629 | 0.022132 |
| WP_ADIPOGENESIS | -1.567644186 | 0.027168629 | 0.022132 |
| WP_EBOLA_VIRUS_PATHWAY_ON_HOST | -1.606287757 | 0.027168629 | 0.022132 |
| WP_FOCAL_ADHESION | -1.474603714 | 0.027168629 | 0.022132 |
| REACTOME_COMPLEMENT_CASCADE | -2.002734836 | 0.027168629 | 0.022132 |
| REACTOME_PARASITE_INFECTION | -2.013991555 | 0.027168629 | 0.022132 |
| KEGG_T_CELL_RECEPTOR_SIGNALING_PATHWAY | -1.539159657 | 0.027168629 | 0.022132 |
| KEGG_TOLL_LIKE_RECEPTOR_SIGNALING_PATHWAY | -1.566886435 | 0.027168629 | 0.022132 |
| REACTOME_INTERLEUKIN_4_AND_INTERLEUKIN_13_SIGNALING | -1.717094189 | 0.027168629 | 0.022132 |
| WP_SPINAL_CORD_INJURY | -1.587759494 | 0.027168629 | 0.022132 |
| REACTOME_BINDING_AND_UPTAKE_OF_LIGANDS_BY_SCAVENGER_RECEPTORS | -2.118601802 | 0.027168629 | 0.022132 |
| WP_HUMAN_COMPLEMENT_SYSTEM | -1.705686985 | 0.027168629 | 0.022132 |
| WP_TOLLLIKE_RECEPTOR_SIGNALING_PATHWAY | -1.557053039 | 0.027168629 | 0.022132 |
| REACTOME_COLLAGEN_FORMATION | -1.717164102 | 0.027168629 | 0.022132 |
| REACTOME_FCERI_MEDIATED_MAPK_ACTIVATION | -2.035852705 | 0.027168629 | 0.022132 |
| WP_ALLOGRAFT_REJECTION | -1.79950433 | 0.027168629 | 0.022132 |
| REACTOME_INTERFERON_GAMMA_SIGNALING | -1.80973006 | 0.027168629 | 0.022132 |
| REACTOME_FCGR3A_MEDIATED_IL10_SYNTHESIS | -2.021517339 | 0.027168629 | 0.022132 |
| REACTOME_INITIAL_TRIGGERING_OF_COMPLEMENT | -2.072748137 | 0.027168629 | 0.022132 |
| KEGG_ECM_RECEPTOR_INTERACTION | -1.720913718 | 0.027168629 | 0.022132 |
| KEGG_HEMATOPOIETIC_CELL_LINEAGE | -1.67128832 | 0.027168629 | 0.022132 |
| REACTOME_INTEGRIN_CELL_SURFACE_INTERACTIONS | -1.750350489 | 0.027168629 | 0.022132 |
| REACTOME_ROLE_OF_PHOSPHOLIPIDS_IN_PHAGOCYTOSIS | -2.039554619 | 0.027168629 | 0.022132 |
| REACTOME_ANTIGEN_ACTIVATES_B_CELL_RECEPTOR_BCR_LEADING_TO_GENERATION_OF_SECOND_MESSENGERS | -1.938226227 | 0.027168629 | 0.022132 |
| REACTOME_FCERI_MEDIATED_CA_2_MOBILIZATION | -1.976550369 | 0.027168629 | 0.022132 |
| KEGG_COMPLEMENT_AND_COAGULATION_CASCADES | -1.740146461 | 0.027168629 | 0.022132 |
| PID_INTEGRIN1_PATHWAY | -1.700303101 | 0.027168629 | 0.022132 |
| REACTOME_FCGR_ACTIVATION | -2.098830422 | 0.027168629 | 0.022132 |
| REACTOME_SCAVENGING_OF_HEME_FROM_PLASMA | -2.159963444 | 0.027168629 | 0.022132 |
| REACTOME_COLLAGEN_BIOSYNTHESIS_AND_MODIFYING_ENZYMES | -1.732709315 | 0.027168629 | 0.022132 |
| REACTOME_COLLAGEN_DEGRADATION | -1.713545181 | 0.027168629 | 0.022132 |
| REACTOME_COSTIMULATION_BY_THE_CD28_FAMILY | -1.680654505 | 0.027168629 | 0.022132 |
| REACTOME_CREATION_OF_C4_AND_C2_ACTIVATORS | -2.112398159 | 0.027168629 | 0.022132 |
| REACTOME_INTERFERON_ALPHA_BETA_SIGNALING | -1.739250293 | 0.027168629 | 0.022132 |
| REACTOME_ROLE_OF_LAT2_NTAL_LAB_ON_CALCIUM_MOBILIZATION | -2.071272862 | 0.027168629 | 0.022132 |
| PID_CD8_TCR_DOWNSTREAM_PATHWAY | -1.815523107 | 0.027168629 | 0.022132 |
| PID_IL12_2PATHWAY | -1.793583344 | 0.027168629 | 0.022132 |
| KEGG_LEISHMANIA_INFECTION | -1.710796789 | 0.027168629 | 0.022132 |
| REACTOME_ASSEMBLY_OF_COLLAGEN_FIBRILS_AND_OTHER_MULTIMERIC_STRUCTURES | -1.696271548 | 0.027168629 | 0.022132 |
| REACTOME_CD22_MEDIATED_BCR_REGULATION | -2.128133003 | 0.027168629 | 0.022132 |
| WP_TYROBP_CAUSAL_NETWORK | -1.733903519 | 0.027168629 | 0.022132 |
| REACTOME_NON_INTEGRIN_MEMBRANE_ECM_INTERACTIONS | -1.688904389 | 0.027168629 | 0.022132 |
| WP_COMPLEMENT_AND_COAGULATION_CASCADES | -1.767221362 | 0.027168629 | 0.022132 |
| REACTOME_CHEMOKINE_RECEPTORS_BIND_CHEMOKINES | -1.707088283 | 0.027168629 | 0.022132 |
| KEGG_AUTOIMMUNE_THYROID_DISEASE | -1.857261501 | 0.027168629 | 0.022132 |
| REACTOME_INTERLEUKIN_10_SIGNALING | -1.785237243 | 0.027168629 | 0.022132 |
| NABA_COLLAGENS | -1.790860871 | 0.027168629 | 0.022132 |
| REACTOME_COLLAGEN_CHAIN_TRIMERIZATION | -1.790860871 | 0.027168629 | 0.022132 |
| REACTOME_AMINE_LIGAND_BINDING_RECEPTORS | -1.808048559 | 0.027168629 | 0.022132 |
| KEGG_TYPE_I_DIABETES_MELLITUS | -1.849936808 | 0.027168629 | 0.022132 |
| WP_MIRNA_TARGETS_IN_ECM_AND_MEMBRANE_RECEPTORS | -1.703145964 | 0.027168629 | 0.022132 |
| KEGG_GRAFT_VERSUS_HOST_DISEASE | -1.844167158 | 0.027168629 | 0.022132 |
| REACTOME_GENERATION_OF_SECOND_MESSENGER_MOLECULES | -1.807162636 | 0.027168629 | 0.022132 |
| WP_TYPE_II_INTERFERON_SIGNALING_IFNG | -1.698697282 | 0.027168629 | 0.022132 |
| KEGG_ALLOGRAFT_REJECTION | -1.852825804 | 0.027168629 | 0.022132 |
| WP_MONOAMINE_GPCRS | -1.836734923 | 0.027168629 | 0.022132 |
| WP_INFLAMMATORY_RESPONSE_PATHWAY | -1.788250152 | 0.027168629 | 0.022132 |
| REACTOME_PD_1_SIGNALING | -1.9201955 | 0.027168629 | 0.022132 |
| REACTOME_SYNDECAN_INTERACTIONS | -1.689791741 | 0.027168629 | 0.022132 |
| WP_CANCER_IMMUNOTHERAPY_BY_PD1_BLOCKADE | -1.779790809 | 0.027168629 | 0.022132 |
| WP_PATHOGENESIS_OF_SARSCOV2_MEDIATED_BY_NSP9NSP10_COMPLEX | -1.79374491 | 0.027270326 | 0.022215 |
| BIOCARTA_CTLA4_PATHWAY | -1.734258171 | 0.027270326 | 0.022215 |
| BIOCARTA_LAIR_PATHWAY | -1.696002113 | 0.027270326 | 0.022215 |
| WP_CELLS_AND_MOLECULES_INVOLVED_IN_LOCAL_ACUTE_INFLAMMATORY_RESPONSE | -1.696002113 | 0.027270326 | 0.022215 |
| BIOCARTA_IL17_PATHWAY | -1.665565025 | 0.027270326 | 0.022215 |
| BIOCARTA_NO2IL12_PATHWAY | -1.775763836 | 0.027270326 | 0.022215 |
| BIOCARTA_CTL_PATHWAY | -1.648258693 | 0.027270326 | 0.022215 |
| BIOCARTA_BLYMPHOCYTE_PATHWAY | -1.705374544 | 0.027270326 | 0.022215 |
| BIOCARTA_TCRA_PATHWAY | -1.649682186 | 0.027270326 | 0.022215 |
| BIOCARTA_TCYTOTOXIC_PATHWAY | -1.688775198 | 0.027270326 | 0.022215 |
| KEGG_PATHWAYS_IN_CANCER | -1.284337697 | 0.037042236 | 0.030176 |
| REACTOME_MAPK_FAMILY_SIGNALING_CASCADES | -1.302529086 | 0.037042236 | 0.030176 |
| WP_PI3KAKT_SIGNALING_PATHWAY | -1.376177663 | 0.037042236 | 0.030176 |
| REACTOME_DEGRADATION_OF_THE_EXTRACELLULAR_MATRIX | -1.52701591 | 0.037042236 | 0.030176 |
| REACTOME_TRANSMISSION_ACROSS_CHEMICAL_SYNAPSES | -1.367951597 | 0.037042236 | 0.030176 |
| WP_IL18_SIGNALING_PATHWAY | -1.408259996 | 0.037042236 | 0.030176 |
| WP_REGULATION_OF_TOLLLIKE_RECEPTOR_SIGNALING_PATHWAY | -1.501808072 | 0.037042236 | 0.030176 |
| WP_CALCIUM_REGULATION_IN_THE_CARDIAC_CELL | -1.474836132 | 0.037042236 | 0.030176 |
| WP_NRF2_PATHWAY | -1.525620341 | 0.037042236 | 0.030176 |
| KEGG_CHEMOKINE_SIGNALING_PATHWAY | -1.417882288 | 0.037042236 | 0.030176 |
| REACTOME_MUSCLE_CONTRACTION | -1.404894388 | 0.037042236 | 0.030176 |
| WP_CILIOPATHIES | -1.416083741 | 0.037042236 | 0.030176 |
| KEGG_LEUKOCYTE_TRANSENDOTHELIAL_MIGRATION | -1.525480354 | 0.037042236 | 0.030176 |
| REACTOME_VISUAL_PHOTOTRANSDUCTION | -1.548015867 | 0.037042236 | 0.030176 |
| WP_GPCRS_OTHER | -1.547540738 | 0.037042236 | 0.030176 |
| WP_PEPTIDE_GPCRS | -1.596570821 | 0.037042236 | 0.030176 |
| PID_AVB3_INTEGRIN_PATHWAY | -1.585052405 | 0.037042236 | 0.030176 |
| KEGG_VIRAL_MYOCARDITIS | -1.585953207 | 0.037042236 | 0.030176 |
| PID_IL4_2PATHWAY | -1.64030731 | 0.037042236 | 0.030176 |
| WP_TCELL_ANTIGEN_RECEPTOR_TCR_PATHWAY_DURING_STAPHYLOCOCCUS_AUREUS_INFECTION | -1.572074954 | 0.037042236 | 0.030176 |
| KEGG_INTESTINAL_IMMUNE_NETWORK_FOR_IGA_PRODUCTION | -1.728739229 | 0.037042236 | 0.030176 |
| PID_SYNDECAN_1_PATHWAY | -1.695137744 | 0.037042236 | 0.030176 |
| PID_NFAT_TFPATHWAY | -1.656037505 | 0.037042236 | 0.030176 |
| REACTOME_ELASTIC_FIBRE_FORMATION | -1.68360288 | 0.037042236 | 0.030176 |
| PID_INTEGRIN3_PATHWAY | -1.61962657 | 0.037042236 | 0.030176 |
| WP_VITAMIN_A_AND_CAROTENOID_METABOLISM | -1.649708576 | 0.037042236 | 0.030176 |
| REACTOME_MET_PROMOTES_CELL_MOTILITY | -1.700015905 | 0.037042236 | 0.030176 |
| WP_MICROGLIA_PATHOGEN_PHAGOCYTOSIS_PATHWAY | -1.623830243 | 0.037042236 | 0.030176 |
| REACTOME_MET_ACTIVATES_PTK2_SIGNALING | -1.69087162 | 0.038075542 | 0.031017 |
| BIOCARTA_TH1TH2_PATHWAY | -1.700454531 | 0.039278922 | 0.031998 |
| BIOCARTA_IL12_PATHWAY | -1.667484348 | 0.039869062 | 0.032478 |
| BIOCARTA_THELPER_PATHWAY | -1.623113242 | 0.041865712 | 0.034105 |
| REACTOME_SEROTONIN_RECEPTORS | -1.595510962 | 0.041865712 | 0.034105 |
| BIOCARTA_MONOCYTE_PATHWAY | -1.587215015 | 0.042127856 | 0.034318 |
| REACTOME_HIGHLY_CALCIUM_PERMEABLE_POSTSYNAPTIC_NICOTINIC_ACETYLCHOLINE_RECEPTORS | -1.588794208 | 0.042127856 | 0.034318 |
| KEGG_SYSTEMIC_LUPUS_ERYTHEMATOSUS | -1.481276347 | 0.048445 | 0.039465 |
| WP_SELENIUM_MICRONUTRIENT_NETWORK | -1.519102744 | 0.048445 | 0.039465 |
| WP_TCELL_ANTIGEN_RECEPTOR_TCR_SIGNALING_PATHWAY | -1.545491034 | 0.048445 | 0.039465 |
| REACTOME_ECM_PROTEOGLYCANS | -1.58658341 | 0.048445 | 0.039465 |
| WP_NONGENOMIC_ACTIONS_OF_125_DIHYDROXYVITAMIN_D3 | -1.518168494 | 0.048445 | 0.039465 |
| PID_TCR_PATHWAY | -1.563525473 | 0.048445 | 0.039465 |
| WP_LUNG_FIBROSIS | -1.558590345 | 0.048445 | 0.039465 |
| REACTOME_SIGNALING_BY_PDGF | -1.556618084 | 0.048474664 | 0.039489 |
| WP_NEPHROTIC_SYNDROME | -1.59670948 | 0.048536745 | 0.039539 |
| WP_PROSTAGLANDIN_SYNTHESIS_AND_REGULATION | -1.602321898 | 0.048536745 | 0.039539 |
| KEGG_TYROSINE_METABOLISM | -1.588366316 | 0.048536745 | 0.039539 |
| REACTOME_VOLTAGE_GATED_POTASSIUM_CHANNELS | -1.581030174 | 0.048536745 | 0.039539 |
| PID_FRA_PATHWAY | -1.648007532 | 0.048541374 | 0.039543 |
| REACTOME_MOLECULES_ASSOCIATED_WITH_ELASTIC_FIBRES | -1.613643017 | 0.048541374 | 0.039543 |
| KEGG_PRIMARY_IMMUNODEFICIENCY | -1.625561106 | 0.048760958 | 0.039722 |
| PID_IL12_STAT4_PATHWAY | -1.667094799 | 0.048989274 | 0.039908 |
| WP_SELECTIVE_EXPRESSION_OF_CHEMOKINE_RECEPTORS_DURING_TCELL_POLARIZATION | -1.662799508 | 0.049062814 | 0.039968 |
| PID_INTEGRIN2_PATHWAY | -1.671765988 | 0.049361932 | 0.040212 |

| **VAX2** | | | |
| --- | --- | --- | --- |
| Description | NES | p.adjust | qvalues |
| REACTOME_G_ALPHA_I_SIGNALLING_EVENTS | -1.746069931 | 0.031047054 | 0.023452 |
| REACTOME_GPCR_LIGAND_BINDING | -1.753388936 | 0.031047054 | 0.023452 |
| REACTOME_NEURONAL_SYSTEM | -1.423437267 | 0.031047054 | 0.023452 |
| REACTOME_NEUTROPHIL_DEGRANULATION | -1.626035346 | 0.031047054 | 0.023452 |
| REACTOME_SIGNALING_BY_INTERLEUKINS | -1.604448726 | 0.031047054 | 0.023452 |
| NABA_CORE_MATRISOME | -1.659997676 | 0.031047054 | 0.023452 |
| NABA_SECRETED_FACTORS | -1.581508506 | 0.031047054 | 0.023452 |
| REACTOME_CLASS_A_1_RHODOPSIN_LIKE_RECEPTORS_ | -1.869857818 | 0.031047054 | 0.023452 |
| REACTOME_LEISHMANIA_INFECTION | -1.716399712 | 0.031047054 | 0.023452 |
| WP_NUCLEAR_RECEPTORS_METAPATHWAY | -1.6040602 | 0.031047054 | 0.023452 |
| KEGG_CYTOKINE_CYTOKINE_RECEPTOR_INTERACTION | -1.767681633 | 0.031047054 | 0.023452 |
| KEGG_MAPK_SIGNALING_PATHWAY | -1.506376876 | 0.031047054 | 0.023452 |
| KEGG_NEUROACTIVE_LIGAND_RECEPTOR_INTERACTION | -1.726668338 | 0.031047054 | 0.023452 |
| REACTOME_PLATELET_ACTIVATION_SIGNALING_AND_AGGREGATION | -1.680979928 | 0.031047054 | 0.023452 |
| REACTOME_TRANSMISSION_ACROSS_CHEMICAL_SYNAPSES | -1.499764409 | 0.031047054 | 0.023452 |
| WP_GPCRS_CLASS_A_RHODOPSINLIKE | -1.830520359 | 0.031047054 | 0.023452 |
| WP_MAPK_SIGNALING_PATHWAY | -1.493769387 | 0.031047054 | 0.023452 |
| KEGG_REGULATION_OF_ACTIN_CYTOSKELETON | -1.569867119 | 0.031047054 | 0.023452 |
| REACTOME_ANTI_INFLAMMATORY_RESPONSE_FAVOURING_LEISHMANIA_PARASITE_INFECTION | -1.77704543 | 0.031047054 | 0.023452 |
| REACTOME_FC_EPSILON_RECEPTOR_FCERI_SIGNALING | -1.554005132 | 0.031047054 | 0.023452 |
| REACTOME_IMMUNOREGULATORY_INTERACTIONS_BETWEEN_A_LYMPHOID_AND_A_NON_LYMPHOID_CELL | -1.86231727 | 0.031047054 | 0.023452 |
| KEGG_CHEMOKINE_SIGNALING_PATHWAY | -1.539870337 | 0.031047054 | 0.023452 |
| REACTOME_G_ALPHA_Q_SIGNALLING_EVENTS | -1.679631334 | 0.031047054 | 0.023452 |
| REACTOME_INTERFERON_SIGNALING | -1.609179317 | 0.031047054 | 0.023452 |
| REACTOME_NEUROTRANSMITTER_RECEPTORS_AND_POSTSYNAPTIC_SIGNAL_TRANSMISSION | -1.517717736 | 0.031047054 | 0.023452 |
| NABA_ECM_GLYCOPROTEINS | -1.719994045 | 0.031047054 | 0.023452 |
| REACTOME_CELL_SURFACE_INTERACTIONS_AT_THE_VASCULAR_WALL | -1.846085169 | 0.031047054 | 0.023452 |
| REACTOME_PEPTIDE_LIGAND_BINDING_RECEPTORS | -1.887166485 | 0.031047054 | 0.023452 |
| WP_VITAMIN_D_RECEPTOR_PATHWAY | -1.525426402 | 0.031047054 | 0.023452 |
| KEGG_CALCIUM_SIGNALING_PATHWAY | -1.701387128 | 0.031047054 | 0.023452 |
| NABA_ECM_AFFILIATED | -1.571064229 | 0.031047054 | 0.023452 |
| REACTOME_FCGAMMA_RECEPTOR_FCGR_DEPENDENT_PHAGOCYTOSIS | -1.747482126 | 0.031047054 | 0.023452 |
| REACTOME_SIGNALING_BY_THE_B_CELL_RECEPTOR_BCR_ | -1.537921128 | 0.031047054 | 0.023452 |
| WP_CILIOPATHIES | -1.499075735 | 0.031047054 | 0.023452 |
| WP_MYOMETRIAL_RELAXATION_AND_CONTRACTION_PATHWAYS | -1.695913972 | 0.031047054 | 0.023452 |
| WP_NRF2_PATHWAY | -1.66434505 | 0.031047054 | 0.023452 |
| KEGG_JAK_STAT_SIGNALING_PATHWAY | -1.714886176 | 0.031047054 | 0.023452 |
| WP_CALCIUM_REGULATION_IN_THE_CARDIAC_CELL | -1.695341664 | 0.031047054 | 0.023452 |
| REACTOME_RESPONSE_TO_ELEVATED_PLATELET_CYTOSOLIC_CA2_ | -1.83931627 | 0.031047054 | 0.023452 |
| KEGG_CELL_ADHESION_MOLECULES_CAMS | -1.676269674 | 0.031047054 | 0.023452 |
| KEGG_LEUKOCYTE_TRANSENDOTHELIAL_MIGRATION | -1.711938296 | 0.031047054 | 0.023452 |
| REACTOME_COMPLEMENT_CASCADE | -1.985930891 | 0.031047054 | 0.023452 |
| REACTOME_PARASITE_INFECTION | -1.812013263 | 0.031047054 | 0.023452 |
| REACTOME_VISUAL_PHOTOTRANSDUCTION | -1.685495597 | 0.031047054 | 0.023452 |
| WP_TOLLLIKE_RECEPTOR_SIGNALING_PATHWAY | -1.727034089 | 0.031047054 | 0.023452 |
| KEGG_T_CELL_RECEPTOR_SIGNALING_PATHWAY | -1.633932988 | 0.031047054 | 0.023452 |
| KEGG_TOLL_LIKE_RECEPTOR_SIGNALING_PATHWAY | -1.713278127 | 0.031047054 | 0.023452 |
| REACTOME_INTERLEUKIN_4_AND_INTERLEUKIN_13_SIGNALING | -1.769758593 | 0.031047054 | 0.023452 |
| REACTOME_BINDING_AND_UPTAKE_OF_LIGANDS_BY_SCAVENGER_RECEPTORS | -1.847721625 | 0.031047054 | 0.023452 |
| REACTOME_FCERI_MEDIATED_MAPK_ACTIVATION | -1.847761979 | 0.031047054 | 0.023452 |
| WP_TCELL_ANTIGEN_RECEPTOR_TCR_SIGNALING_PATHWAY | -1.73572648 | 0.031047054 | 0.023452 |
| REACTOME_ANTIGEN_ACTIVATES_B_CELL_RECEPTOR_BCR_LEADING_TO_GENERATION_OF_SECOND_MESSENGERS | -1.763725701 | 0.031047054 | 0.023452 |
| REACTOME_FCERI_MEDIATED_CA_2_MOBILIZATION | -1.952283161 | 0.031047054 | 0.023452 |
| REACTOME_INTERFERON_GAMMA_SIGNALING | -1.796067764 | 0.031047054 | 0.023452 |
| WP_ALLOGRAFT_REJECTION | -1.852054393 | 0.031047054 | 0.023452 |
| WP_SELENIUM_MICRONUTRIENT_NETWORK | -1.696546786 | 0.031047054 | 0.023452 |
| REACTOME_FCGR3A_MEDIATED_IL10_SYNTHESIS | -1.865051636 | 0.031047054 | 0.023452 |
| WP_HUMAN_COMPLEMENT_SYSTEM | -1.921029024 | 0.031047054 | 0.023452 |
| REACTOME_ROLE_OF_PHOSPHOLIPIDS_IN_PHAGOCYTOSIS | -1.833082929 | 0.031047054 | 0.023452 |
| KEGG_HEMATOPOIETIC_CELL_LINEAGE | -1.860356545 | 0.031047054 | 0.023452 |
| REACTOME_INITIAL_TRIGGERING_OF_COMPLEMENT | -1.951374476 | 0.031047054 | 0.023452 |
| WP_PEPTIDE_GPCRS | -1.793397037 | 0.031047054 | 0.023452 |
| KEGG_COMPLEMENT_AND_COAGULATION_CASCADES | -1.837678667 | 0.031047054 | 0.023452 |
| REACTOME_COSTIMULATION_BY_THE_CD28_FAMILY | -1.778695598 | 0.031047054 | 0.023452 |
| REACTOME_FCGR_ACTIVATION | -1.90517055 | 0.031047054 | 0.023452 |
| REACTOME_SCAVENGING_OF_HEME_FROM_PLASMA | -1.86904952 | 0.031047054 | 0.023452 |
| REACTOME_CREATION_OF_C4_AND_C2_ACTIVATORS | -1.931136101 | 0.031047054 | 0.023452 |
| KEGG_LEISHMANIA_INFECTION | -1.816367741 | 0.031047054 | 0.023452 |
| PID_CD8_TCR_DOWNSTREAM_PATHWAY | -1.817634175 | 0.031047054 | 0.023452 |
| REACTOME_CD22_MEDIATED_BCR_REGULATION | -1.864619302 | 0.031047054 | 0.023452 |
| WP_TYROBP_CAUSAL_NETWORK | -1.988227001 | 0.031047054 | 0.023452 |
| PID_IL12_2PATHWAY | -1.805250628 | 0.031047054 | 0.023452 |
| WP_COMPLEMENT_AND_COAGULATION_CASCADES | -1.777542726 | 0.031047054 | 0.023452 |
| REACTOME_CHEMOKINE_RECEPTORS_BIND_CHEMOKINES | -1.753625841 | 0.031047054 | 0.023452 |
| KEGG_AUTOIMMUNE_THYROID_DISEASE | -1.922443786 | 0.031047054 | 0.023452 |
| REACTOME_INTERLEUKIN_10_SIGNALING | -1.947908666 | 0.031047054 | 0.023452 |
| REACTOME_AMINE_LIGAND_BINDING_RECEPTORS | -1.843015095 | 0.031047054 | 0.023452 |
| KEGG_TYPE_I_DIABETES_MELLITUS | -1.836518043 | 0.031047054 | 0.023452 |
| REACTOME_GAP_JUNCTION_ASSEMBLY | -1.74516752 | 0.031047054 | 0.023452 |
| KEGG_ALLOGRAFT_REJECTION | -1.834812088 | 0.031047054 | 0.023452 |
| KEGG_GRAFT_VERSUS_HOST_DISEASE | -1.773950866 | 0.031047054 | 0.023452 |
| REACTOME_GENERATION_OF_SECOND_MESSENGER_MOLECULES | -1.914846427 | 0.031047054 | 0.023452 |
| WP_TYPE_II_INTERFERON_SIGNALING_IFNG | -1.736483214 | 0.031047054 | 0.023452 |
| WP_MONOAMINE_GPCRS | -1.909715767 | 0.031047054 | 0.023452 |
| WP_INFLAMMATORY_RESPONSE_PATHWAY | -1.812283851 | 0.031047054 | 0.023452 |
| WP_SELECTIVE_EXPRESSION_OF_CHEMOKINE_RECEPTORS_DURING_TCELL_POLARIZATION | -1.742928089 | 0.031047054 | 0.023452 |
| BIOCARTA_INFLAM_PATHWAY | -1.732645328 | 0.031047054 | 0.023452 |
| KEGG_ASTHMA | -1.818275977 | 0.031047054 | 0.023452 |
| PID_INTEGRIN2_PATHWAY | -1.796452568 | 0.031047054 | 0.023452 |
| PID_TCR_CALCIUM_PATHWAY | -1.716876473 | 0.031047054 | 0.023452 |
| REACTOME_PD_1_SIGNALING | -1.982896171 | 0.031047054 | 0.023452 |
| WP_IL1_AND_MEGAKARYOCYTES_IN_OBESITY | -1.870671102 | 0.031047054 | 0.023452 |
| WP_CANCER_IMMUNOTHERAPY_BY_PD1_BLOCKADE | -1.769660039 | 0.031047054 | 0.023452 |
| BIOCARTA_LAIR_PATHWAY | -1.845177821 | 0.031047054 | 0.023452 |
| WP_CELLS_AND_MOLECULES_INVOLVED_IN_LOCAL_ACUTE_INFLAMMATORY_RESPONSE | -1.845177821 | 0.031047054 | 0.023452 |
| WP_PLATELETMEDIATED_INTERACTIONS_WITH_VASCULAR_AND_CIRCULATING_CELLS | -1.751368356 | 0.031047054 | 0.023452 |
| BIOCARTA_NO2IL12_PATHWAY | -1.793728583 | 0.031047054 | 0.023452 |
| BIOCARTA_ASBCELL_PATHWAY | -1.705385501 | 0.031047054 | 0.023452 |
| WP_CELLTYPE_DEPENDENT_SELECTIVITY_OF_CCK2R_SIGNALING | -1.689370434 | 0.031047054 | 0.023452 |
| BIOCARTA_BLYMPHOCYTE_PATHWAY | -1.823601322 | 0.031047054 | 0.023452 |
| BIOCARTA_TCRA_PATHWAY | -1.657929991 | 0.031047054 | 0.023452 |
| BIOCARTA_TCYTOTOXIC_PATHWAY | -1.769662505 | 0.031047054 | 0.023452 |
| BIOCARTA_THELPER_PATHWAY | -1.763828852 | 0.031047054 | 0.023452 |
| REACTOME_EXTRACELLULAR_MATRIX_ORGANIZATION | -1.483700868 | 0.04194619 | 0.031685 |
| WP_FOCAL_ADHESIONPI3KAKTMTORSIGNALING_PATHWAY | -1.374105901 | 0.04194619 | 0.031685 |
| REACTOME_SLC_MEDIATED_TRANSMEMBRANE_TRANSPORT | -1.419511685 | 0.04194619 | 0.031685 |
| KEGG_FOCAL_ADHESION | -1.489093176 | 0.04194619 | 0.031685 |
| REACTOME_BIOLOGICAL_OXIDATIONS | -1.456844923 | 0.04194619 | 0.031685 |
| REACTOME_MUSCLE_CONTRACTION | -1.488181838 | 0.04194619 | 0.031685 |
| WP_FOCAL_ADHESION | -1.450332525 | 0.04194619 | 0.031685 |
| WP_METAPATHWAY_BIOTRANSFORMATION_PHASE_I_AND_II | -1.485671567 | 0.04194619 | 0.031685 |
| REACTOME_ION_CHANNEL_TRANSPORT | -1.485428516 | 0.04194619 | 0.031685 |
| REACTOME_TOLL_LIKE_RECEPTOR_CASCADES | -1.529937535 | 0.04194619 | 0.031685 |
| WP_REGULATION_OF_TOLLLIKE_RECEPTOR_SIGNALING_PATHWAY | -1.65830893 | 0.04194619 | 0.031685 |
| KEGG_NATURAL_KILLER_CELL_MEDIATED_CYTOTOXICITY | -1.604981642 | 0.04194619 | 0.031685 |
| REACTOME_STIMULI_SENSING_CHANNELS | -1.598502219 | 0.04194619 | 0.031685 |
| WP_GPCRS_OTHER | -1.609431078 | 0.04194619 | 0.031685 |
| KEGG_FC_GAMMA_R_MEDIATED_PHAGOCYTOSIS | -1.594702195 | 0.04194619 | 0.031685 |
| REACTOME_INTEGRIN_CELL_SURFACE_INTERACTIONS | -1.655180019 | 0.04194619 | 0.031685 |
| REACTOME_INTERFERON_ALPHA_BETA_SIGNALING | -1.762147289 | 0.042018144 | 0.031739 |
| REACTOME_ROLE_OF_LAT2_NTAL_LAB_ON_CALCIUM_MOBILIZATION | -1.762315433 | 0.042018144 | 0.031739 |
| WP_TCELL_ANTIGEN_RECEPTOR_TCR_PATHWAY_DURING_STAPHYLOCOCCUS_AUREUS_INFECTION | -1.702268397 | 0.042018144 | 0.031739 |
| PID_NFAT_TFPATHWAY | -1.755559682 | 0.042574541 | 0.032159 |
| KEGG_INTESTINAL_IMMUNE_NETWORK_FOR_IGA_PRODUCTION | -1.860841589 | 0.042574541 | 0.032159 |
| PID_UPA_UPAR_PATHWAY | -1.669858905 | 0.042574541 | 0.032159 |
| WP_VITAMIN_A_AND_CAROTENOID_METABOLISM | -1.688883283 | 0.042574541 | 0.032159 |
| REACTOME_DAG_AND_IP3_SIGNALING | -1.651595956 | 0.042574541 | 0.032159 |
| WP_FIBRIN_COMPLEMENT_RECEPTOR_3_SIGNALING_PATHWAY | -1.703501932 | 0.042574541 | 0.032159 |
| WP_MICROGLIA_PATHOGEN_PHAGOCYTOSIS_PATHWAY | -1.736572844 | 0.042635659 | 0.032205 |
| KEGG_PRIMARY_IMMUNODEFICIENCY | -1.772513757 | 0.042703091 | 0.032256 |
| BIOCARTA_TH1TH2_PATHWAY | -1.745491279 | 0.045 | 0.033991 |
| WP_PATHOGENESIS_OF_SARSCOV2_MEDIATED_BY_NSP9NSP10_COMPLEX | -1.760656109 | 0.045 | 0.033991 |
| BIOCARTA_CTLA4_PATHWAY | -1.754593847 | 0.045226992 | 0.034163 |
| REACTOME_REGULATION_OF_TLR_BY_ENDOGENOUS_LIGAND | -1.720599268 | 0.045290787 | 0.034211 |
| BIOCARTA_IL5_PATHWAY | -1.665228288 | 0.047891156 | 0.036175 |
