# Supplementary figures and images for: Protective Prognostic Biomarkers Negatively Correlated with Macrophage M2 Infiltration in Low-Grade Glioma
Source: J Oncol. 2022 Apr 8;2022:3623591. doi: 10.1155/2022/3623591 (PMC9012619; doi:10.1155/2022/3623591)

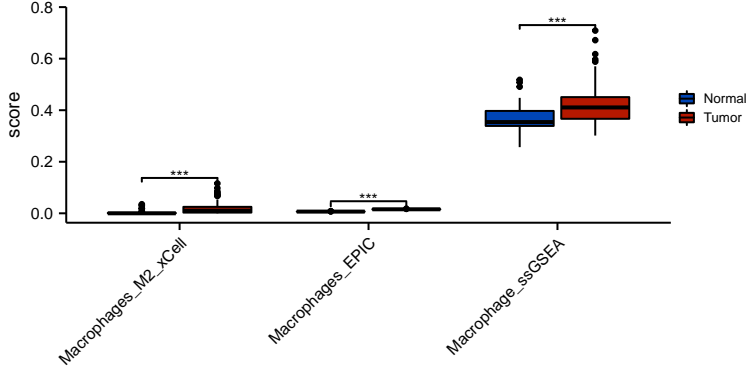

Supplement: Supplementary Materials — Supplementary file 1: clinical information of the datasets. Supplementary file 2: results of GSEA analysis. Figure S1: results of xCell, EPIC, and ssGSEA algorithms about Macrophage. [file 3623591.f1.zip › FigureS1.pdf]
